# Radiocarbon dating and microarchaeology untangle the history of Jerusalem's Temple Mount: A view from Wilson's Arch

**DOI:** 10.1371/journal.pone.0233307

**Published:** 2020-06-03

**Authors:** Johanna Regev, Joe Uziel, Tehillah Lieberman, Avi Solomon, Yuval Gadot, Doron Ben-Ami, Lior Regev, Elisabetta Boaretto

**Affiliations:** 1 D-REAMS Radiocarbon Laboratory, Scientific Archaeology Unit, Weizmann Institute of Science, Rehovot, Israel; 2 Israel Antiquities Authority, Jerusalem, Israel; 3 The Department of Land of Israel Studies and Archaeology, Bar Ilan University, Ramat Gan, Israel; 4 Independent scholar, Ramat Gan, Israel; 5 The Lester and Sally Entin Faculty of Humanities, Tel Aviv University, Tel-Aviv, Israel; University at Buffalo - The State University of New York, UNITED STATES

## Abstract

Radiocarbon dating is rarely applied in Classical and Post-Classical periods in the Eastern Mediterranean, as it is not considered precise enough to solve specific chronological questions, often causing the attribution of historic monuments to be based on circumstantial evidence. This research, applied in Jerusalem, presents a novel approach to solve this problem. Integrating fieldwork, stratigraphy, and microarchaeology analyses with intense radiocarbon dating of charred remains in building materials beneath Wilson's Arch, we absolutely dated monumental structures to very narrow windows of time–even to specific rulers. Wilson’s Arch was initiated by Herod the Great and enlarged during the Roman Procurators, such as Pontius Pilatus, in a range of 70 years, rather than 700 years, as previously discussed by scholars. The theater-like structure is dated to the days of Emperor Hadrian and left unfinished before 132–136 AD. Through this approach, it is possible to solve archaeological riddles in intensely urban environments in the historical periods.

## Introduction

The eye-catching classical architecture in the Mediterranean Basin was constructed by or during the reigns of historically documented figures. Other than the plain pleasure of knowing which stone belongs to whose days, these structures can fill in gaps in our understanding of historical processes and events, that is, when correctly dated. Surprisingly though, in most cases, the dating of the monuments has been based on material culture correlations, coins, and texts, whereas radiocarbon dating has rarely been applied to date urban architectural complexes. Where constructional timbers have been found, dendrochronology is a useful tool [e.g., 1–3]. However, such finds are limited, due to the climatic conditions of the Mediterranean Basin, while well-built stone monuments stand the test of time, being used and reused over centuries, making their dating highly challenging. Such is the situation at Wilson’s Arch, located at the heart of Jerusalem’s old city, outside the walls of the Temple Mount.

**Fig 1:**
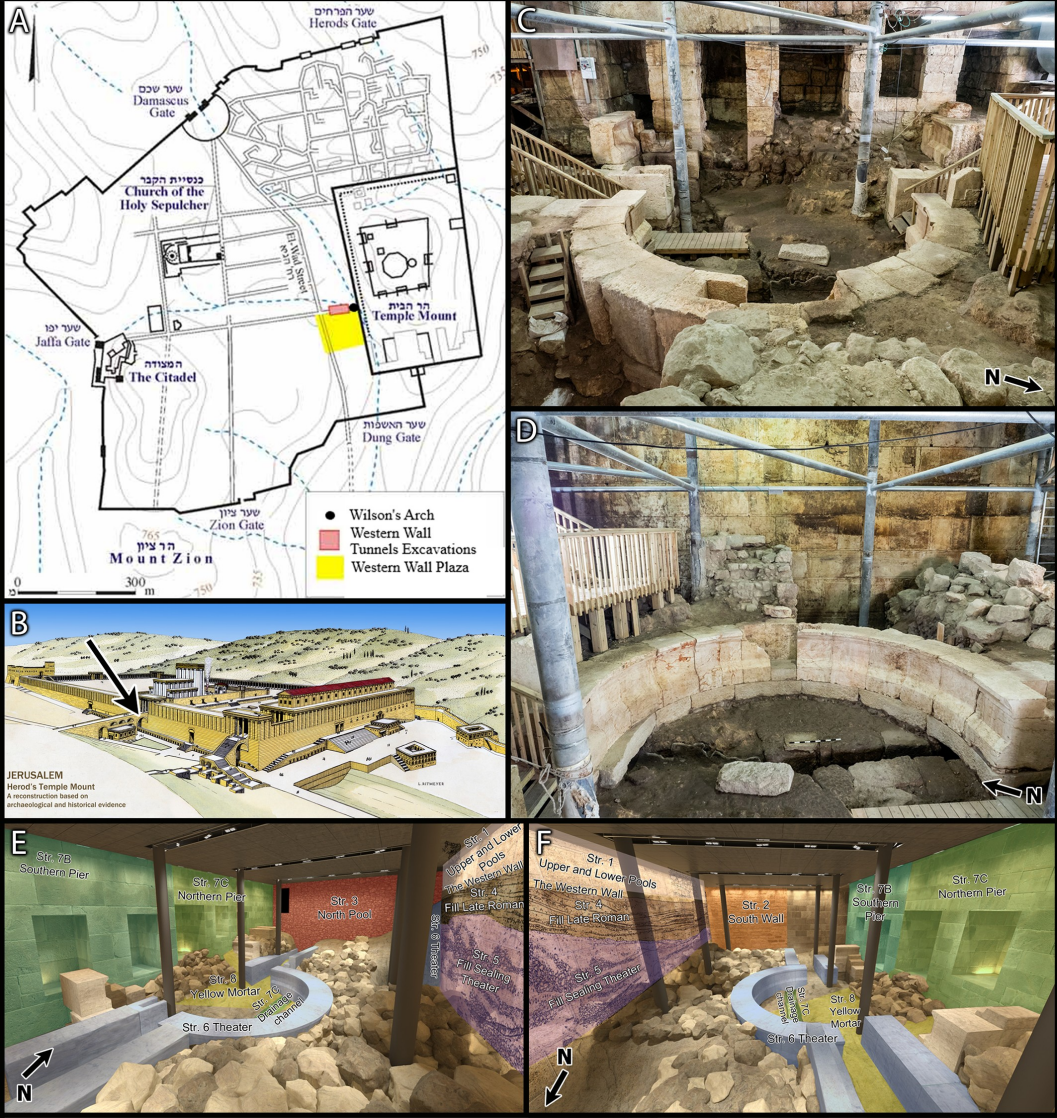
Wilson’s Arch excavation area. (A) Map of the old city of Jerusalem and the location Wilson’s Arch. Copyrights: Israel Antiquities Authority, 2020. (B) An artistic reconstruction of the Temple Mount in the time of Herod the Great (1st century AD). The arrow points to the arch known today as Wilson's Arch. Copyrights: Ritmeyer Archaeological Design, 2020. (C,D) Photographs of the site. The scale bar in D is 1 meter in length. (E,F) A 3D reconstruction of the site. As the site is under constant renovations, a model is used here to illustrate the location of the various features and strata. A section drawing of strata 1,4,5 was imposed on the Western Wall to illustrate their relative position.

Wilson’s Arch is a large easternmost arch of a long bridge, approximately 100 m in length, dubbed “The Great Causeway” built of superimposed arches leading from the west to the Temple Mount, which enabled easy access over the Tyropeon Valley in the past, and linked between the houses of Jerusalem's "Upper City" to the Temple Mount (Fig 1). It is of no surprise then that Wilson’s arch and the Great Causeway have been the subject of research for over 170 years [4,5,6, Fig 1]. Scholars have debated the dating of this monument over the years, with spanning from the time of Herod the Great, through the Roman colonization of the city, and up to the early Islamic period. From 2015 to 2019, an archaeological excavation was undertaken beneath the arch, as part of tourist development, and to provide a chronological dating for the arch itself. In order to date the dense architectural sequence exposed, including Wilson’s Arch, an intensive ^14^C dating project was undertaken during the excavation, integrating simultaneous stratigraphic and microarchaeological analysis. This allowed for detailed radiocarbon sampling while the context was still *in-situ* and high precision for the radiocarbon dating of the construction of the various monuments exposed. Consequently, this research allowed for the study of these remains on the backdrop of the historical setting in which they were constructed. The main features uncovered (and radiocarbon dated) in the excavations span from the late Hasmonean period (100–50 BC) through the Mamluk period (1400 AD), consisting of layers of fill, walls, pools, channels, a theater-like structure, and the different building stages of the arch. The latter two are of particular importance in the milieu of Jerusalem in times of extreme political and cultural change, with tensions between the local Jewish culture and the encroaching Roman world. The correct timing of the features exposed in the excavations furthers our understanding of the effects that historical events had on Jerusalem.

The strategy used here for radiocarbon dating began in the field, through stratigraphic analysis, the relationship of the material for dating and the sediments, in order to select the most pristine context. This needs to be done while excavating and requires the application of microarchaeology based on the use of analytical methods (e.g., microscope, chemistry and spectroscopy) to study the archaeological context characteristics that are not visible through the naked eye and are essential for high precision and accuracy of radiocarbon dating [7,8].

It should be noted that in order to reduce the introduction of noise into the chronological framework (e.g., outliers), the radiocarbon sampling methodology was tailored according to the context and material in the field. In this manner, the most secure stratigraphic contexts were used for radiocarbon dating. As such noise has multiple origins (e.g., chemical contamination by environmental or anthropogenic activities, redeposition); the sampling is best done at the same time as the features are exposed. The real-time sampling is necessary in order to sample the surrounding sediments for establishing the mineralogical differences and confirm the pristine and distinct nature of the dated archaeological context in comparison with the material from other contexts or layers.

In ancient plaster and mortar preparation, charred remains were often incorporated as additives. However, single seeds in mortars, which are the best material for high-resolution chronology, are often very fragile due to diagenesis [9]. As a single seed of wheat is enough for radiocarbon dating, it is essential to recognize such objects in the field as they may later deteriorate once removed from the archaeological context. Only in this way is it possible to increase the number of dates on a single entity, avoiding the need to combine several seeds.

Another issue that could only be overcome by immediate sampling was preservation works, which had to be undertaken rapidly at the site. As part of these works, modern bonding materials mixed with archaeological sediments were used in order to fill gaps between the stones, making any further sampling for radiocarbon dating impossible and highly risky due to contamination from these materials.

## Materials and methods

All samples were taken during the salvage excavations conducted by the authors, under the permission and authority of the Israel Antiquities Authority (Permit Numbers 7514, 7633, 7900, 8205). All samples used in this research are stored at the Kimmel Center for Archaeological Science (Weizmann Institute of Science, Rehovot, Israel).

The Wilson’s arch excavations were systematically sampled for ^14^C dating during the entire excavation, in full cooperation between the field archaeologists and the radiocarbon specialists. As new significant features were unearthed, the best ways to accurately date the features were investigated in the field and laboratory. As many of the features uncovered in the area were walls and structures without remaining intact floors, construction materials, such as plaster coating constructed elements and mortar found between stones, were collected, and the charred remains found inside the construction materials were dated.

The dated samples consisted of a single piece of charred material; in most cases, short-lived material such as seeds, twigs, and grasses. As this material type represents a single growth year, it was given a higher priority for dating. We did not find any large constructional timbers or charcoal fragments with an identifiable outer ring and multiple rings, which could have been used for dendrochronology or wiggle matching tree ring sequences. Therefore, the small charcoal fragments that were found could have originated from the inner parts of branches and trunks or even older construction beams. In such cases, their dating would yield earlier dates relative to the construction event (e.g., the ‘old wood effect’). Hence, charcoal was considered for dating only in cases where short-lived material was not found. To attain enough charred material for dating after pretreatment, we preferred to begin with at least 3 mg of charred material. Since the charred pieces in many of the contexts would break or turn into powder by the touch of a tweezer, and even more so when collected into a sediment bag or sieved, the method used was often to collect each piece of charred material into a separate aluminum envelope already in the field. The surface of the sediment chosen for dating was properly cleaned, and the sediment was carefully isolated (see S1 Data for more details). Sediment samples for FTIR analyses using Nicolet iS5 (Thermo) FTIR instrument in 4 cm^–1^ resolution were taken from the context intended for dating, as well as from adjoining sediment, which was used as control samples, in order to characterize the nature of the sediments using proxies such as burnt clay [10], phosphate [8], and disordered calcite, based on the grinding curves method [11]. Botanical identification of the samples was undertaken using the binocular microscope SMZ 800N (Nikon) and the metallurgical microscope eclipse LV150N (Nikon).

The samples were pretreated for ^14^C dating using the acid-base-acid (ABA) protocol [9,12] and measured at the Dangoor Research Accelerator Mass Spectrometer (D-REAMS) at the Weizmann Institute [13]. The ^14^C ages were calibrated using the OxCal software version 4.3.2 [14] according to the IntCal13 atmospheric curve [15] (see S2 Table in S1 Data).

The OxCal models (S6 Standard model, S7 Outlier model and S3 Table in S1 Data) were built as a sequence of phases based on stratigraphy. The boundaries between phases were contiguous when no gaps were expected between the two phases. When a significant elapse of time between two phases was expected, a sequential boundary was used. An overlapping boundary was used in Str. 7C between the phases “Northern pier of the arch” and “Northern drainage channel,” as they may have been built simultaneously or the channel may have been built slightly later. The mortars and plasters from a single architectural phase had, in general, very similar ages (e.g., mathematically their average pass the χ^2^-square test), suggesting that mainly freshly carbonized material was used for construction purposes.

### Standard model

For this model, 33 dates were used. Three residual dates and four dates from unclear strata were excluded. This model gave a model Agreement of 90% (minimum agreement for a model is 60%).

### Outlier model

For this model, 36 dates were used, with the four dates from unclear strata excluded. With the Outlier Model [16], three samples were identified as outliers. One sample (RTD-9378) was from a brown fill, which also contained residual pottery and turned out also to be residual. Another sample (RTD-9301) originated from Wall 4493, the “yellow mortar” (see S1 Data). The third residual measurement (RTD-9331) came from construction mortar, where charcoal was used as an integral component of the mortar. In this case, the sample was only slightly too old for the model and could be explained as an analytical outlier or that the mortar contained burned remains of a slightly older fire event.

We preferred, given our detailed methodology in the field for the sample collection, to remove the three outliers from the model and run a Standard Model, following the suggestion by Bronk Ramsey et al. [16], as “probably the best approach” once the outliers can be securely identified.

We noticed an earlier shift of ~10 years in the modeled calibrated years of Stratum 7 between the ‘Standard Model’ without the outliers and the ‘Outlier Model,’ due to sample RTD-9331 (S3 Table in S1 Data). In accordance with Bronk Ramsey’s recommendation above, we consider the prior removal of the outliers in the model preferable.

## Results

### *In-situ* context verification through the application of microarchaeology

Great care was taken in defining the stratigraphy and verifying the archaeological context of the collected samples. We found that microarchaeological tools in general, and especially Fourier-transform infrared spectroscopy (FTIR) are most useful in achieving this aim, as various anthropogenic activities altered the sediments in the past, leaving different mineralogical signatures in them [8]. Accordingly, the FTIR analysis was performed on the building materials and other archaeological sediments from which the charred material for dating was collected (S3 Table, S1 Table, S1 Fig in S1 Data). Where possible, later deposited sediments covering the contexts and structures to be dated were sampled in order to compare and verify the different nature of the building materials and these sediments.

**Fig 2:**
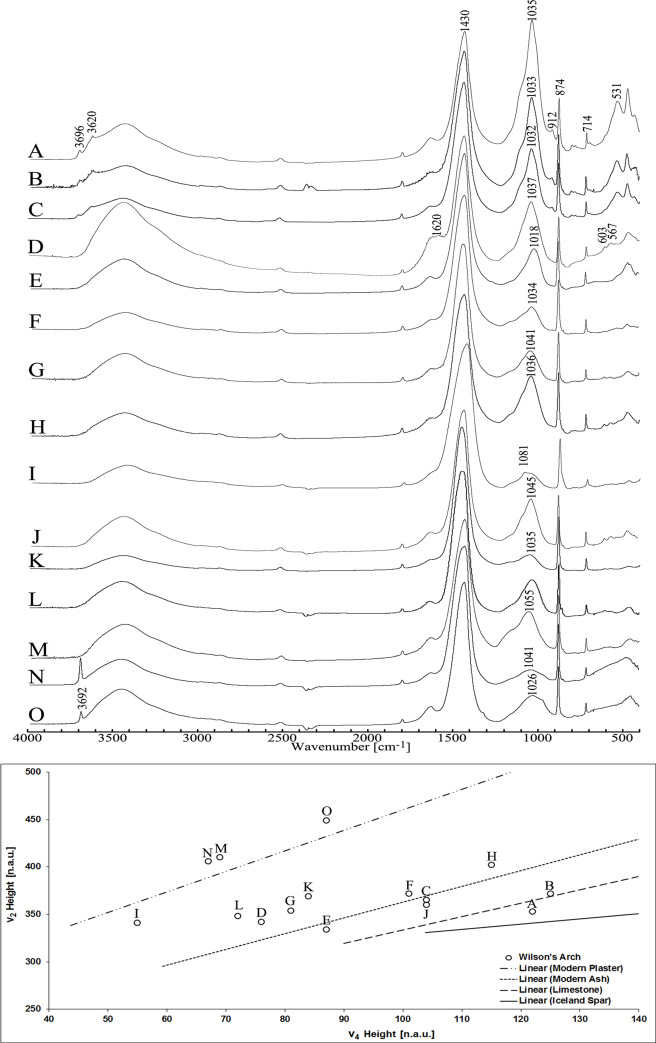
Sediments FTIR spectra and “Grinding curves” plot. Top: FTIR spectra of the materials correlating to Table 1. Bottom: “Grinding curves” plot (after Regev et al. [11], with modifications), illustrating the differences in the local atomic order of the calcitic fraction of the spectra above (circles), compared to standard geogenic and pyrogenic calcite minerals (linear lines). The vicinity of the samples to specific lines point to the probable origin of the calcite fraction in the sample. The FWHM values of chalk and ash samples are noted below and above their trend lines, respectively.

Most of the materials analyzed in the excavated area belong to construction materials, represented by mortar or plaster of which the main component is calcite (see in S1 Data, and figures therein for stratigraphic and context details). The most characteristic FTIR spectra of the sample types are given in Fig 2 (top), while the location of the calcite component of the samples on the ‘grinding curve’ plot, characteristic of the calcite origin and preservation state of the samples [11], is presented in Fig 2 (bottom). Based on the FTIR spectra, the ‘fill’ samples (Fig 2 and 2A–2C) are the only ones to contain unaltered clay fraction (based on the presence of peaks at 3696 and 3620 cm^-1^), they do not contain phosphate minerals (no peaks at 603 and 567 cm^-1^), and the calcite fraction is in the region of the limestone-chalk trendlines. The fill/dump layer, which yielded high quantities of burnt seeds (spectrum D), is of very different nature, having all the proxies of heat altered sediments. The plasters, on the other hand (Fig 2 and 2K–2O), contain heat-altered clays (no peaks at 530, 3696 and 3620 cm^-1^), a calcite fraction closer or equivalent to the modern plaster trendline, and even a Ca(OH)_2_ carbonation in some plasters (Fig 2, 2N and 2O, peak at 3692 cm^-1^), indicating an incomplete setting process. The mortars between the stones (Fig 2 and 2G–2J) are more varied in nature, but nevertheless can be clearly distinguished from overlying fills by either a shift in the location of the clay peak (towards wavenumbers higher than 1036 cm^-1^), the presence of phosphate or the location of the calcite peak on the grinding curve in or between the trendlines of ash and plaster.

The importance of the microarchaeological method applied is demonstrated in the contrast between the fill sediments covering the building materials, as the fills penetrated into the crevices between the hewn stones, with color and texture not too different than the mortars. Clearly, dating charred remains from such fills would give an incorrect, intrusive age for the structure, or at best, a *terminus ante quem* for its use. Even if the differences were recognizable by the naked eye in the field, the application of FTIR gave significant verification for the initial identification in the field through the use of an independent analytical method.

### Radiocarbon dating of the various strata

Below is a short description of the archaeological framework of the dated contexts (Fig 1, see supporting information for additional archaeological and microarchaeological information and analysis), combined with the results obtained through radiocarbon measurements for the dating of the specific layers.

**Fig 3:**
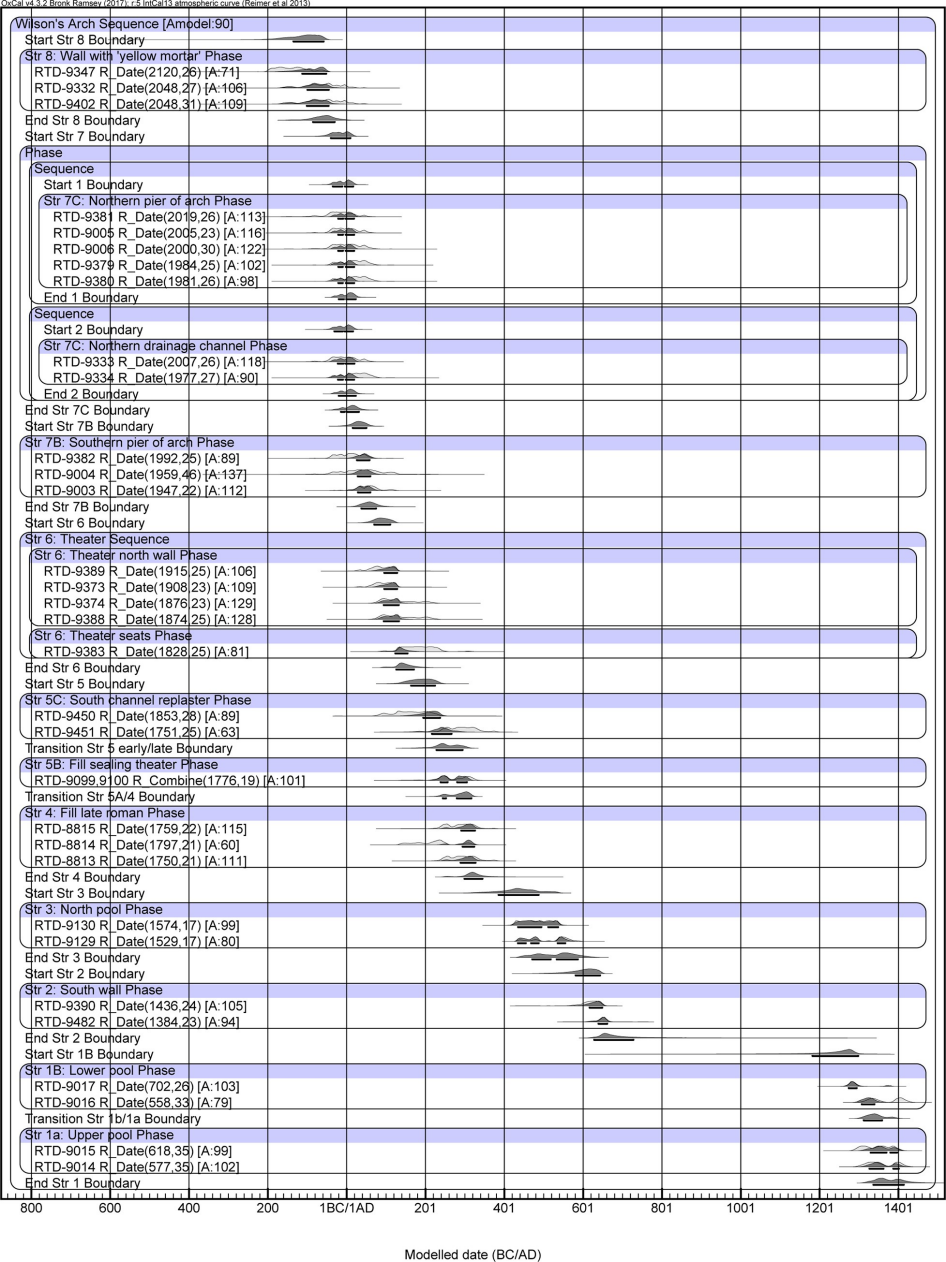
Multiplot of the stratigraphy-based radiocarbon model of Wilson’s arch. The areas plotted in black depict the modeled posterior age of the sample, while the light gray areas depict the entire calibrated range of the measurement.

A total of 40 samples were measured (see Table 1 for the modeled ranges and S2 Table for the uncalibrated and calibrated unmodeled ranges in S1 Data). Of these, four (RTD 9219, 9220, 9217 and 9218) were excluded from the analysis, despite their fitting the general chronological scheme, as they could not be securely attributed to a stratigraphic phase. Three samples (RTD 9301, 9378, and 9331) yielded dates that were too early (residual and below 60% agreement value in the Standard Bayesian model, See S3 Table in S1 Data) and were also excluded. Therefore, the final stratigraphic model (Standard Model) consists of 33 samples (Fig 3). The calibrated ranges throughout the paper refer to the ±1σ probability distribution (68.2%). The modeled results are presented in Table 1 (For unmodeled data see S2 Table in S1 Data) For clarity, we summarized the results of each phase in the column “Most Probable Age,” so that all dates were rounded to the nearest interval of 5 years (e.g., 0, 5, 10, etc.). Where all dates were nearly identical, we simply used the rounded figures (Str 7C, 7B, 6, 5B, 4, 3, 1A). In cases where the dates within a phase varied (Str 8, 5C, 2, 1B), we used the OxCal query “Last” (see S1 Data) to determine the most probable age. We considered this query to be most accurate since some of the charred material included in the building materials could have been burned sometime before they were incorporated into the building material, albeit it seems that the majority of the dates depict a time close to the construction. The agreement of the Standard Model is 90% (Fig 3).

**Table 1:** Radiocarbon dating results after Bayesian modeling of the different strata. The modeled age ranges are presented in Fig 3. See the explanation above for how the most probable age ranges of the layers were determined.

Model Stratum	Most Probable Age	Sample Lab Code	Modelled (BC/AD) 68.2%	Agreement of Sample	FTIR Figure
Str 8: Wall with “yellow mortar“	90–45 BC	RTD-9347	114–53 BC	71.2	F
		RTD-9332	101–46 BC	105.4	
		RTD-9402	102–46 BC	108.8	
Str 7C: Northern pier of arch—mortar	20 BC-20 AD	RTD-9381	22BC-20 AD	112.4	I
		RTD-9005	22BC-20 AD	116.3	
		RTD-9006	22BC-20 AD	121.5	
		RTD-9379	22BC-20 AD	102.2	
		RTD-9380	22BC-20 AD	97.6	
Str 7C: Northern drainage channel—plaster	20 BC-20 AD	RTD-9333	23 BC-20 AD	117.5	K
		RTD-9334	22BC-20 AD	90.7	
Str 7B: Southern pier of arch—mortar	30–60 AD	RTD-9382	27–59 AD	88.5	J
		RTD-9004	29–61 AD	136.7	
		RTD-9003	29–61 AD	112.5	
Str 6: Theater north wall—mortar	95–135 AD	RTD-9389	96–129 AD	105.9	H
		RTD-9373	96–129 AD	108.7	
		RTD-9374	95–133 AD	128.5	
		RTD-9388	95–133 AD	128.1	
Str 6: Theater seats—fill	125–155 AD	RTD-9383	124–155 AD	80.6	B
Str 5C: South channel replaster—plaster	220–265 AD	RTD-9450	195–238 AD	88.9	L
		RTD-9451	217–266 AD	62.3	
Str 5B: Fill sealing theater	240–305 AD	RTD-9099,9100 R_Combine	239–306 AD	101.1	Similar to D
Str 4: Fill Late Roman	290–330 AD	RTD-8815	291–326 AD	114.4	D
		RTD-8814	294–324 AD	60.4	
		RTD-8813	289–328 AD	110.5	
Str 3: North pool—plaster	435–555 AD	RTD-9130	435–537 AD	99.4	M
		RTD-9129	435–555 AD	79	
Str 2: South wall—mortar	640–660 AD	RTD-9390	617–649 AD	104.9	G
		RTD-9482	642–663 AD	93.7	
Str 1B: Lower pool—plaster	1305–1340 AD	RTD-9017	1275–1295 AD	102.7	O
		RTD-9016	1307–1340 AD	78	
Str 1A: Upper pool—plaster	1330–1400 AD	RTD-9015	1330–1397 AD	99.4	N
		RTD-9014	1327–1402 AD	101.8	
Control sediments for FTIR and samples excluded from the model					
Str. 4 and 5 fill	Fill inside the southern pier of the arch (B. 107)				A
Str. 6 fill	Fill next to the north wall of the (B. 424393) theater, under the seats				B
Str 4. fill	Fill inside the southern channel (B. 423735)				C
Unknown Str. mortar	“yellow mortar” at the entrance to the theater (B. 423581)				E

The total statistical agreement of the OxCal model is 90%. The letters in the column “FTIR Figure” corresponds to the FTIR spectra in Fig 2. These refer to the sediment from which the samples were taken from. At the bottom of the table, samples of the fill sediments adjacent to the dated contexts appear.

#### Str 8

(see also S3 Fig in S1 Data): The earliest feature exposed in the excavation was a massive, 14 meters thick, element of stones in various sizes, embedded in the yellow mortar (Wall 4493) with very little charred remains included (see also S1 Data). The feature had been exposed in the past, extending towards the west, and attributed to the Hasmonean Period (late 2^nd^-early 1^st^ centuries BC), and may have functioned as a dam or wall [see e.g., 17]. This element served as the foundation for the features built at later times–both the various stages of the pier of Wilson's Arch and the theater-like structure (see further discussion below). Of the four samples measured, only three dated the structure (RTD 9347, 9332, 9402) and one, RTD 9301 appears to be residual (see S2 Table in S1 Data). The measured samples gave ranges between 200BC-1AD. The modeled results of those three samples range between 115–45 BC with a most probable age of 90–45 BC.

#### Str 7

(see also S3-S5 Figs in S1 Data): Above the Stratum 8 wall, the pier of Wilson's arch was constructed. Three distinct phases were noted in the pier (Str. 7): the earliest phase consisted of the north half of the pier (7.4 m wide), which was likely part of an earlier bridge leading into the Temple Mount with a single room at its center. The second phase doubled the width of the bridge (14.8 m wide), with two additional integrated rooms, and at a third stage, two more rooms were breached in the northern pier (Fig 1C and 1E). Historically, the pier and the arch are of great significance, as they provided access to the Temple Mount. Prior to the current excavation, opinions regarding the dating of the arch spanned some 700 years, beginning with the Herodian period and up to the Ummayad period [for a summary of the opinions regarding the date of the arch, see, e.g., 18]. The lack of clear surfaces related to each of the stages made it difficult to date these features using standard archaeological relative dating. In order to date the various phases, samples were taken from the mortar relating to the various stages. Five samples were collected in order to date the northern pier: RTD 9381 from the ceiling of the northernmost room (S4 Fig in S1 Data); RTD 9006 and 9005 from the original, central room (S3 Fig in S1 Data); and RTD 9379 and 9380 from beneath the pier (S3 Fig in S1 Data).

For the southern pier, four dates were obtained: RTD 9003 and 9004 from the ceiling and walls of one of the rooms in the pier (S4 Fig in S1 Data); and RTD 9331 and RTD 9382 from mortar beneath the pier (S4 Fig in S1 Data).

Also attributed to Stratum 7 were two phases of a drainage channel. The northern phase cuts Wall 4493 (the “yellow mortar” of Str. 8) and appears to be the earlier of the two. The channel utilizes the yellow mortar of the earlier wall, which was cut and then covered with plaster, while the southern part was built of stone and resembles the Early Roman drainage channel, discovered further to the south [e.g., 19] beneath the main street leading to the Temple Mount [20]. Two dates (RTD 9333 and 9334, S5 Fig in S1 Data) were determined from the charred seeds in the plaster covering the northern channel, and two samples (RTD 9450 and 9451, S5 Fig in S1 Data) from short-lived charred material inside the mud-plaster, found covering the joins between the stones of the southern channel. It is important to note that the plaster of the southern channel likely belongs to the final use of the channel during the late Roman period and not its construction in the Early Roman period. Recently, data from the excavations to the south, near the Pool of Siloam, have shown that the drainage channel mentioned above continued in use in the Late Roman period [21].

The building of the northern arch and the northern drainage channel (stratum 7C), modeled with an overlapping boundary for the two features, has a lower likelihood (20%) between 22–11 BC and higher likelihood (49%) between 3 BC-20 AD. The southern expansion of the arch (Str. 7B) gives ranges between 30–60 AD.

#### Str 6

(see also S6 Fig in S1 Data): Overlying the drainage channel, a small theater-like structure was built, constructed between the Western Wall of the Temple Mount and the pier of the arch (Fig 1D, 1E and 1F). It is the first such structure found in Jerusalem, likely belonging to the Roman occupation of the city after the destruction of 70 AD. It is important to note that the period following the destruction of Jerusalem and until the later stages of Roman Jerusalem in the 4^th^ century AD is often treated as a single archaeological phase, despite the many important historical events that occur in the region, particularly the establishment of the military camp of the Tenth Roman Legion Fretensis (70 AD), the declaration of the colony ‘Aelia Capitolina’ (~130 AD), the outbreak of the Second Jewish Revolt (132–135 AD, although its effects on Jerusalem were likely limited), the death of Emperor Hadrian (138 AD) and the exit of the Tenth Roman Legion Fretensis from the city (~285 AD). Regardless, the ceramic dating obtained from probes in the foundations of the theater places its construction somewhere in the 2^nd^ century AD [18]. The historical events within the first half of the 2^nd^ century AD are of great significance to the development of the city under Roman hegemony. Therefore, the possibility to narrow this dating to a more specific time contributes to our understanding of the site. In order to narrow down the dating, four samples were taken from the mortar between stones in the northern wall of the theater (RTD 9389, 9373, 9374 and 9388 see S6 Fig in S1 Data), representing the early stages of construction, while two additional dates (RTD 9383 and 9378 see S6 Fig in S1 Data) were taken from the construction fill used to support the theater seats. The four dates, measured from the north wall of the theater, are very uniform in age, and the modeled ranges fall between 95–135 AD. The modeled date range of the sample of the fill between the theater seats yields a range between 125–155 AD.

#### Str 5

(see also S7 Fig in S1 Data): It appears that after the theater-like structure went out of use, several archaeological events occurred. First, the southern channel from Str. 7 was replastered (Str. 5C), although shortly after both it and the theater-like structure were covered over by a series of fills, composed of thick layers of sediment (Str. 5B). The reason for the dumping of the fills cannot be ascertained, although their ceramic and numismatic dating place these levels in the late 3^rd^ century AD, putting the theater out of use completely. This was likely done in order to reorganize the urban layout of this area at that time. Samples were dated from the fills. The later date from the southern channel (Str. 5C), understood to represent re-plastering rather than the initial building time, ranges between 220–265 AD. The dates (RTD 9099, 9100, S7 Fig in S1 Data) from the fill of Str. 5B exhibit two distributions, (30%) between 240–257 AD and (38%) 280–306 AD.

#### Str 4

(see also S7 Fig in S1 Data): Stratum 5 was sealed by a striation of deposits of sediment that were dumped beneath the arch. While the purpose of this intentional filling remains unclear, the ceramic and numismatic dating places these levels in the 4^th^ century AD. Some of these layers consisted almost purely of charred olive pits and ash. The unusual amounts of olive pits suggest that organized dumping activities were taking place at the foot of the Temple Mount. Superimposed samples were dated from the fills (RTD 8813, 8814, and 8815), yielding a date range between 290–330 AD.

#### Str 3

(see also S8 Fig in S1 Data): Along the northern boundary of the excavation, the wall of a large cistern was exposed. Although the cistern had been exposed in the past [17:222–241], its dating had remained enigmatic. A section excavated in its southern wall provided the opportunity to date the cistern's construction. Two samples (RTD 9129 and 9130) were analyzed. The dates have a wide range of possible dates, spanning from 435–555 AD.

#### Str 2

(see also S8 Fig in S1 Data): The southern boundary of the excavation was formed by two walls–the western one, which was part of a Byzantine structure (Str. 3), and the eastern one, which abuts the Byzantine structure and the Western Wall. This wall had no related surfaces, although it cut the Strata 4 and 5 fills. It was therefore attributed to Stratum 2 and dated by two samples of mortar taken between the stones (RTD 9390, 9482). The dates of this context range between 640–660 AD.

#### Str 1

(see also S7 Fig in S1 Data): The uppermost layer exposed in the excavation was a large plastered pool, built beneath the arch. Two distinct phases of the pool were noted, each built of a layer of plaster overlying a cement-like layer, approximately 0.5 m thick. The pool–known as ‘Al-Burak*’–*continued to exist until modern times. Four samples were measured in order to date the different stages of the pool (RTD 9016 and 9017 for the earlier pool–Str. 1B; RTD 9014 and 9015 for the later pool–Str. 1A). The early pool (Str. 1B) was built between 1305–1340 AD. Its second phase (Str. 1A) was reconstructed between 1330–1400 AD.

## Discussion

**Fig 4:**
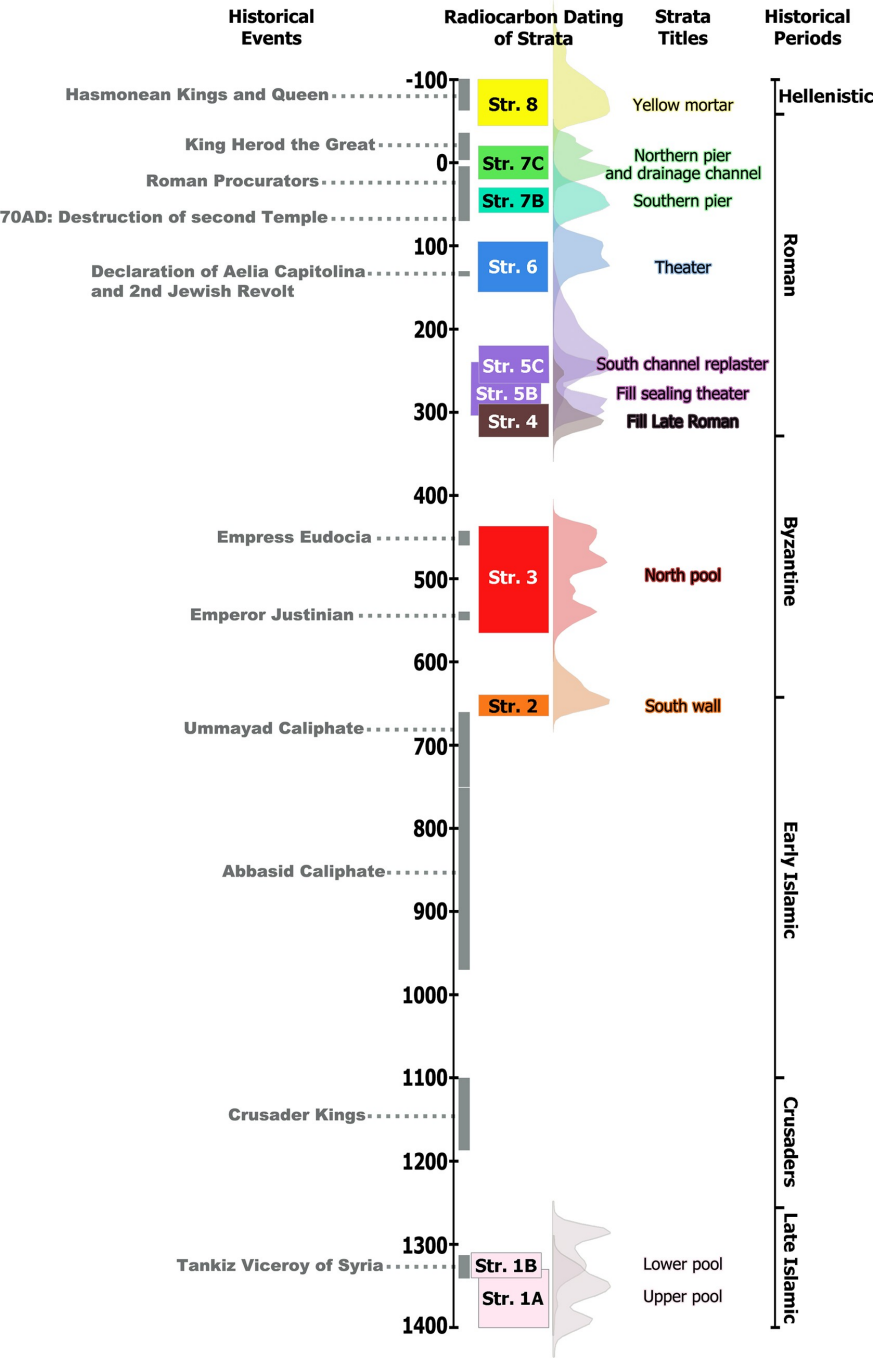
Summarizing chronological chart of Wilson’s arch excavation. Comparing the rulers and major events in the history of Jerusalem to the radiocarbon dating of the strata. The grey vertical rectangles mark the length of the historical events. The histograms represent the total probability distributions of the radiocarbon measurements of each stratum (using the 'Sum' function in OxCal).

The absolute dating of the features exposed beneath Wilson's Arch, coupled with the integration of microarchaeological analysis for the context and detailed on-site sampling for radiocarbon dating allowed for high precision attribution of the elements exposed and their position in the historical reconstruction of Jerusalem (Fig 4), particularly at the western foot of the Temple Mount. Dating short-lived organic materials from the construction mortars and plasters proved to be a suitable method for attaining a reliable chronology in a dense urban setting. The practical working method was to identify potential building materials and extract single seeds from them in the field as the charred remains were very fragile. Micro-archaeological tools (especially FTIR) were used for verification of the samples as construction materials. This proved to be of crucial importance, as the dating of the arch itself was the *raison d'être* of the excavation, and had been largely debated, with the possibility of dating through standard archaeological methods to a period of some 700 years [e.g., 4,17,18]! Lacking *in-situ* floor contexts abutting the arch, the only possibility to refine the date of this feature–of utmost importance to the urban network of Jerusalem's center on the Temple Mount–was through the secure definition of construction mortar and its dating. The resolution achieved through Bayesian modeling of the dates, coupled with stratigraphic analysis, narrowed the dating from seven centuries to two independent stages of construction of fewer than 50 years each. The first stage was constructed based on the radiocarbon dates, during the reign of Herod the Great, or slightly after his death. This was an early stage in the expansion of the Temple Mount, which was initiated, but possibly not completed by Herod the Great [see, e.g., 19,22]. In the second stage, the arch was expanded, creating a bridge that was 14.8 m wide, which still stands today in its original form, accessing the Temple Mount in its final dimensions. Dating the expansion to the period of 30–60 AD implies that the entire arch, known today as Wilson’s arch, was built before the Great Jewish Revolt beginning in 66 AD [5]. The expansion of the bridge in the early part of the 1^st^ century AD coincides with major building activities that were undertaken in Jerusalem during the time of the Roman procurators [23].

Among these projects were the paving of a central street and the amendment of a water aqueduct supplying the Temple Mount with water [23]. The dates from Wilson's Arch support the premise that although Herod the Great had begun major construction projects, particularly in the vicinity of the Temple and the Temple Mount, these activities continued after his death under direct Roman rule in the city, including the construction of a complex street network, which included the path that was supported by Wilson's arch, leading from the upper city to the Temple Mount.

Of no less importance are the events–and buildings uncovered–after the destruction of Jerusalem in 70 AD, at which time the Tenth Roman Legion Fretensis and its veterans occupied the city, eventually leading to the declaration of the Roman colony ‘Aelia Capitolina’ by Emperor Hadrian. As part of this process, a small theater-like structure (Str. 6) was constructed beneath Wilson's Arch. Interestingly, several features in the building indicate that the structure was not complete [18]. The lack of completion led to a similar situation as described above, with the need to define construction mortars and securely date them. As the dating falls within the range of 95–135 AD, the onset of the theater's construction was most likely initiated before the outbreak of the Second Jewish Revolt in 132 AD (also known as the Bar Kochva revolt). Although mundane reasons for the incompletion of the building, such as lack of funds, cannot be ruled out completely, it is possible that significant historical events could be behind this. These primarily include two events deeply affecting Jerusalem and its surroundings: The Second Jewish Revolt and the death of Hadrian (138 AD). Regardless, the dating of the construction of this structure to the days around the declaration of Aelia Capitolina provides information about the development and character of the colony, which included civic buildings, such as the theater-like structure uncovered here.

The theater remained uncovered, used, or unused for about a century, at least until the re-plastering of the southern channel took place (Str. 5C), between the years 220–265 AD. The earliest dumping activities (Str. 5B) covering the theater are dated to 240–305 AD. After the theater-like structure was sealed by the fills of Stratum 5, the area continued to be used for extensive dumping of ash-filled with olive pits dated to 290–330 AD, likely originating from industry or bathhouse located nearby.

It is important to note that the excavation methodology aimed at ^14^C dating of the complete sequence of settlement beneath the arch. Therefore, many other important dates were retrieved. The construction of the earliest feature exposed (Str. 8, “yellow mortar” of wall 4493) was dated to the period between 90–45 BC, i.e., the end of the Hasmonean period. Archaeological excavations further to the west exposed additional portions of this wall, which considering its thickness and solid construction, may have been part of the fortifications known as the First Wall, undertaken during the rule of the Hasmonean kings. Nearby segments of the First Wall were both built and later repaired during that period, whereas the construction of this feature in the area of Wilson’s Arch may have been executed during the reign of Alexander Jannaeus, who is known from various historical sources to be involved in the expansion of the Hasmonean kingdom (e.g., the book of Maccabees, Josephus *AJ* 16:65–73). Although there is some doubt as to the extent of the historicity of these accounts, the dating of Stratum 8 to the early 1^st^ century BC provides independent grounds to support the scenario that Jerusalem would have been fortified in this period by the "First Wall" (and see 7 for further discussion and references therein).

Wilson’s Arch used this solid feature as its foundation and for 2000 years has witnessed changes of rulers and urbanism in Jerusalem. Within these, one can note the large water reservoir built adjacent to the arch (Str. 3), which extended to the north of the excavation area. This reservoir was built during the Byzantine period, between 435–555 AD. During this period, the city witnessed extensive construction, including the paving of the Western Cardo and founding of the Nea Church. These dates regrettably fall at a time where the calibration curve hits a plateau, where different years result in similar radiocarbon value measured today due to fluctuations in past atmospheric ^14^C concentrations. This calibration plateau does not allow for a more precise dating without multiple stratigraphically sequenced dates or dendrochronology samples, not to be found at the site.

On the south side of the excavation area, a wall standing to a height of 4 m was exposed (Str. 2). The date of this wall falls on a precise part of the radiocarbon calibration curve, dating it to the Early Islamic period, between 640 to 660 AD, soon after the Muslim conquest of Jerusalem in 637 AD. From this time, until the rule of the Mamluks, dateable buildings or layers were not found, as they may have been removed by the final and impressive building project of the large pools beneath Wilson’s Arch. An interesting feature of these pools was the excellent state of preservation of organic remains of straw and flax fibers inside the plasters, allowing us to date cellulose of this short-lived material. The FTIR analyses revealed the presence of the mineral brucite (Mg(OH)_2_), recently patented as a nontoxic preservative due to its antibacterial and antifungal properties [24]. It remains for future research to reveal if these properties were already known to the Mamluk builders and whether this material could contribute to the quality of the water in cisterns. The earlier pool dates between 1305–1340 AD, at the time of Tankiz, viceroy of Syria, a range within which many construction projects were undertaken throughout the city and specifically to the west of the Temple Mount, including the Tankiziyya and the Madrasa [17].

## Conclusions

The integration of radiocarbon experts in archaeological fieldwork, alongside the use of microarchaeology during the archaeological excavation of Wilson's Arch, has made it possible to resolve one of the most intriguing riddles in archaeology related to the construction of this architectural feature. The construction of the initial bridge leading to the Temple Mount, as determined by radiocarbon between 20 BC and 20 AD, can now be associated with Herod the Great. Later, between 30 AD and 60 AD, the bridge doubled in size. These events, both taking place in the Early Roman period, reflect on the importance of this feature in the cityscape and on the constant state of construction in Jerusalem, as noted in the works of Flavius Josephus (*BJ*, *AJ*). The only event which caused the cease of the constant construction, reconstruction and expansion of the temple platform and its surroundings was the outbreak of the revolt against Rome and Jerusalem's demise. The construction of a theater-like structure, radiocarbon dated to the rule of Hadrian, indicates that the area surrounding the Temple Mount was the focus of intentional building activities revolving around Roman culture and it is possible that a second revolt–or *The Second Revolt* in 132 AD–is what led to the ceased construction, as supported by the new radiocarbon dates. No less important than the specific structures dated, and their historical significance is the way in which this data was obtained. To date, Jerusalem–like many other historical cities, particularly of the classical period and late antiquity–has suffered from a lack of absolute dating, primarily due to the methods used in the excavations of such cities. The grand monuments often lead to an architectural focus, with precise dating difficult due to the lack of datable finds and the long, extended use and reuse of such structures. By using a large number of samples in a small excavation area, coupled with stratigraphic analysis, and intentionally searching for charred material in mortar and plaster within the structures for their dating, we overcame such problems, dating both the construction and cessation of use of elements such as Wilson's Arch itself–still standing today, yet built almost 2,000 years ago. Such a method can certainly be more broadly applied in densely built areas—in Jerusalem and other ancient cities—to provide much more fine-tuned dating of the remains.

## Supporting information

S1 Data (DOCX)
