# Supplementary material for: Radiocarbon dating and microarchaeology untangle the history of Jerusalem's Temple Mount: A view from Wilson's Arch
Source: PLoS One. 2020 Jun 3;15(6):e0233307. doi: 10.1371/journal.pone.0233307 (PMC7269203; doi:10.1371/journal.pone.0233307)
Supplement: S1 Data — (DOCX) [file pone.0233307.s001.docx]

**S1 Field sampling strategy**

A close co-operation with the site archaeologists and the radiocarbon research team was practiced during the entire excavation, enabling the sampling of most of the features in the field by the radiocarbon team. The collection locations of the samples were marked by basket numbers and photographed. Multiple places in one dated context were sampled, in order to verify the mineralogical uniformity of the sediments of the context and for reproducibility of the dated samples.

This decision to date only single pieces (meaning not to combine several charred fragments for one radiocarbon sample), preferably of short-lived material, affected the working methodology, as the charred material in our studied area was often very fragile and in small pieces. When it was possible to identify the charred material in the field as a seed, it was marked on the aluminum envelope and later verified in the lab. This was done since sometimes the seed would break into powder inside the envelope, making the identification in the laboratory difficult. In contexts where the charred material was better preserved, a stack of botanical sieves was used for collecting the charred material, on-site and/or in the laboratory. If the sediments were wet, they were collected into bags as lumps, dried in the air, and the charred material was recovered under binocular in the laboratory.

The various sampling methodologies used, depending on the sample type is summarized below. The type of the material was defined by its constructional use, rather than the chemical composition.

**Mortar** *(RTD-9381, 9005, 9006, 9379, 9380, 9331, 9382, 9004, 9003, 9389, 9373, 9374, 9388, 9217, 9218,* 9390, 9482, 9017, 9015*)*. In many cases, *in situ* mortar could be found, used between the building stones as part of the building process in the past. First, the outer face of the mortar was cleaned and pictured. If the material did not seem clean enough, the outer layer was scraped out and discarded. If all the material seemed clean, the first layer was collected to the about 2-3cm depth and marked as “outer layer,” and then the deeper layer was collected, and material for dating was preferably sought within it, as it is found deeper between the stones.

**“Yellow mortar” underlying the buildings** *(*RTD-*9301, 9347, 9402, 9332, 9219, 9220*). The yellow material underlying the theater and rooms supporting Wilson’s Arch was sampled in several locations for dating. Since this material was very scarce in pottery, charred remains or bones, much effort was invested in finding samples large enough for dating. In the field, the area was well cleaned exposing clean yellow material, then, using dental tools under intense light, small single pieces of charred remains were collected separately.

**Plaster** (RTD-9333, 9334, 9450, 9451, 9130, 9129, 9016, 9014). With the term ‘plaster’ in this study we refer to the coating material on top of stones and between cracks either for purely aesthetic purposes or to make the surface impermeable to water. For dating plaster, the organic materials within the plaster were recovered (the plaster mineral phase itself was not dated). The plaster was cleaned and removed as chunks. The chunks of plaster were carefully searched for organic remains under binocular in the laboratory.

**Stratified sediment layers** *(RTD-9099, 9100, 8814, 8815, 8813)*. The stratified sediment layers overlaying the theater and partly filling the rooms were sampled. Charred materials and sediments were collected by carefully separating between each layer that could be identified. When possible, samples chosen for dating were taken from directly superimposed layers. When the samples were not directly superimposed, careful attention was given for stratigraphy, to enable reliable stratigraphic modeling for the layers. Most of the material was collected by sieving the specific sediments of the separate layers.

**Constructional fill** (RTD-9378, 9383). In order to narrow down the dating of the theater, some of the brown fills underneath the theater, and the theater’s benches were sampled. The fill contained pottery from pre-Iron Age till the 2^nd^ century AD, and accordingly, the dates could only serve as *Terminus Post Quem* dates.

**S2 Sample selection for radiocarbon chronology**

Samples were selected and measured in several stages. At first, one or two samples were measured from a key context. Based on the results, more samples were chosen from either the same context or contexts relating to it. When a sample was dated to a period with short calibration ranges (a slope in the calibration curve), more samples were chosen from the same context to reach maximum precision for the context. In the cases where the calibrated ranges were large (a plateau in the calibration curve), more effort was invested for attaining multiple contexts relating to each other, in order to perform stratigraphic Bayesian modeling to narrow down the calibrated ranges.

**S3 Fourier Transform Infrared spectroscopy (FTIR) analysis**

The on-site sediment sampling was particularly directed towards radiocarbon dating. Sediment samples for mineralogical analysis using FTIR were collected from all the dated contexts, and when relevant, control samples were taken from the covering fills in order to verify the different nature of the fill materials and the building materials. Several (2-10) FTIR analyses were performed of each context, and the most representative spectra are shown in Fig 2.

**Sample preparation:** Sediments for FTIR analysis were prepared as follows: a few milligrams of sediment were crushed in an agate mortar and pestle. A small amount of KBr was added, mixed, and pressed under 2 tons to form a 7-mm pellet. The pellet was then measured with a Nicolet iS5 (Thermo) FTIR instrument (32 scans, 4 cm^–1^ resolution).

**Spectrum interpretation:** Heat alteration of the clay fraction was estimated according to Berna et al. (17). Differences in the possible origin of the calcite fraction were identified according to the ‘grinding curves’ method (9). In short, calcite minerals, formed through geogenic (spar, limestone, chalk) or pyrogenic (ash, lime plaster) processes possess different local atomic order of the crystal. That difference is expressed in the height ratios of the FTIR absorbance peaks of the mineral and can be used to identify different sources of calcite in the various samples.

Although chalk and ash have very similar grinding curve position, the values of the full width at half maximum (FWHM) of the main absorbance peak (around 1430 cm^-1^) are different and can sometimes be used as a guide to differentiate between them (Fig 2).

Mineral identification was performed using the Kimmel Center for Archaeological Science FTIR reference library (available online at <http://www.weizmann.ac.il/kimmel-arch/infrared-spectra-library>).

**S4 Collected samples for analysis and their archaeological context**

**Table S1. List of samples collected, their types (such as plaster, mortar), and archaeological contexts.**

| **S1 Fig** | **Stratum and function** | **Context description** | **Samples dated of similar material type** |
| --- | --- | --- | --- |
| A | Str. 4 and 5 Fill | B.107 Fill inside the southern pier of the Arch | - |
| B | Str. 6 Fill | B.424393 Fill next to the north wall of the theater, under the seats | RTD-9378*  RTD-9383 |
| C | Str. 4 Fill | B.423735 Fill inside the southern channel | - |
| D | Str. 5 Fill/dump | Fill/striations of roman dumps full of olive pits | RTD-8815  RTD-8814  RTD-8813 |
| E | unknown Str. Mortar | B.423581“Yellow Mortar” at the entrance to the theater | RTD-9219  RTD-9220 |
| F | Str. 8 Mortar | Representative sediment of the “Yellow Mortar” in various places | RTD-9301*  RTD-9347  RTD-9332  RTD-9402 |
| G | Str. 2 Mortar | B.136 Mortar in the South wall | RTD-9482 |
| H | Str. 6 Mortar | B.424395 Mortar in the north wall of the theater | RTD-9389  RTD-9373  RTD-9374  RTD-9388 |
| I | Str. 7C Mortar | B.118 Mortar in the north pier of the arch | RTD-9381  RTD-9005  RTD-9006  RTD-9379  RTD-9380 |
| J | Str. 7B Mortar | B.103 Mortar in the south pier of the arch | RTD-9331*  RTD-9382  RTD-9004  RTD-9003 |
| K | Str. 7C Plaster | B.423737 Plaster of the north channel | RTD-9333  RTD-9334 |
| L | Str. 5C Plaster | B.423736 Plaster of the south channel | RTD-9450  RTD-9451 |
| M | Str. 3 Plaster | B.420222 Plaster of the north pool | RTD-9130  RTD-9129 |
| N | Str. 1A Plaster | L.4213 Plaster of the lower (earlier) pool | RTD-9015  RTD-9014 |
| O | Str. 1B Plaster | L.4215 Plaster of the upper (later)pool | RTD-9017  RTD-9016 |

The samples are arranged according to their types and not stratigraphically. Samples marked with asterisk gave residual ages and were not included in the final model. The letters (first column) refer to the corresponding photos in S1 Fig and to the Fig 2 in main manuscript infrared spectra.


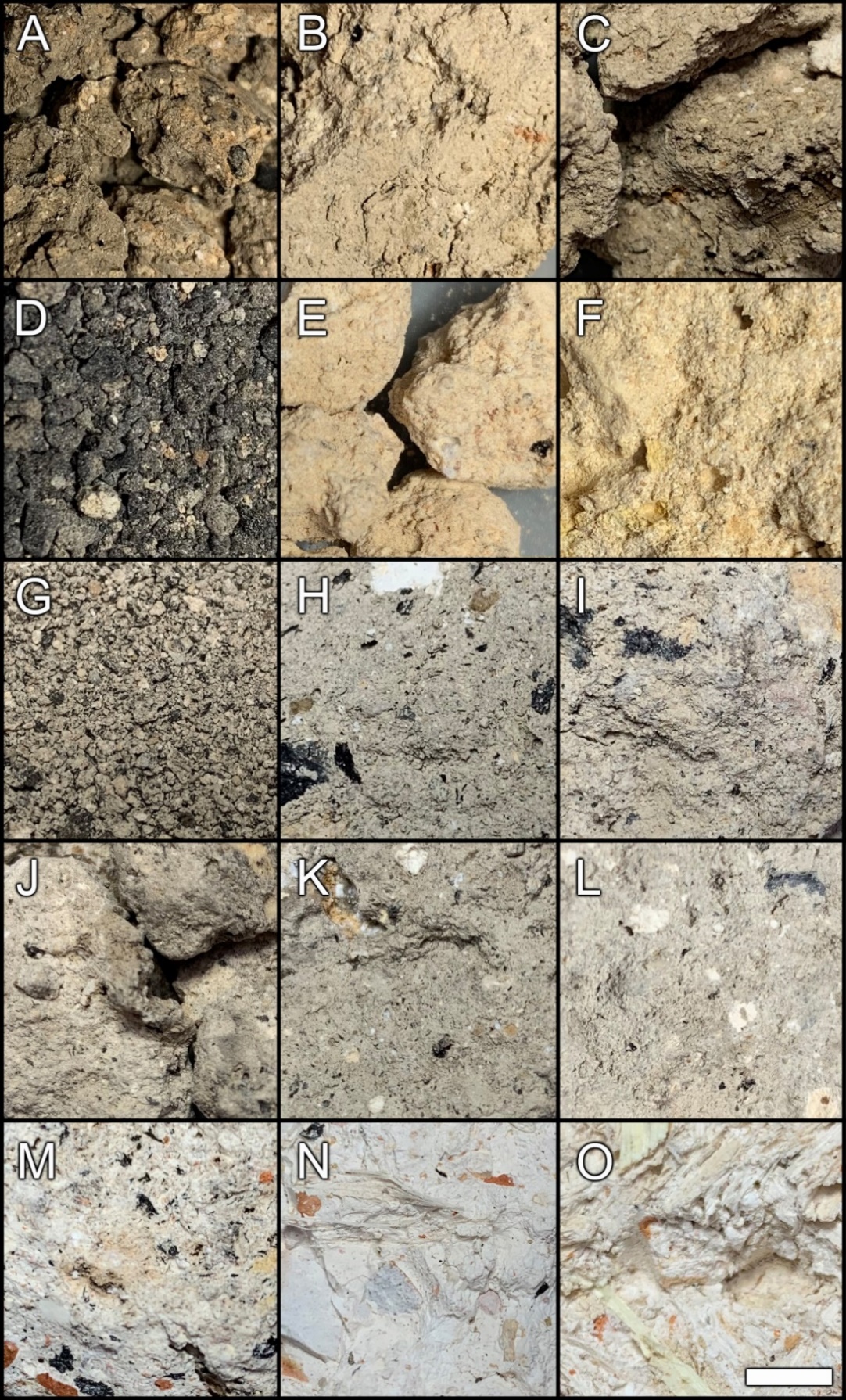


**Fig S1. Images of the various material types sampled.** Photographed pictures under uniform lighting of the materials from which samples were taken for dating, and nearby fill sediments used for comparison of the different mineral composition. The annotation is after Table 1. Scale bar is 0.5 cm.

**S5 FTIR Analysis and detailed contexts of ^14^C samples**

**“Yellow mortar” of wall 4493, Str. 8**

The “yellow mortar” of Wall 4493 was sampled in multiple places (Fig 1, S2 Fig), both for dating and for microarchaeological analysis. In this material the charred remains were very meager indicating that there was no intentional addition of charred material for the construction of this feature. This observation is supported by the abundant presence of charred material in all the other mortars. The FTIR spectra of this sediment appears mostly uniform across sampled locations (Fig 2 F, S1 Fig F), the major component being calcite of geogenic origin based on the grinding curves method, the samples falling on the same location as limestone (not shown) and chalk (FWHM = 110 cm^-1^). A small quantity of clay was present, and the main peak of the clay was located at 1030-1034 cm^-1^. At the north entrance corridor into the theater, different spectra were identified. The location of the clay peak was at 1018-1021 cm^-1^, being unusually low for the local clays (Fig 2 E, S1 Fig E). This is significant, since the two seeds measured from this specific context gave slightly later ages than the samples measured from all the remaining areas. Even though the samples taken from this corridor originated from carefully isolated seeds from within the yellow substance after removal of a 20 cm size stone, the mineralogical composition difference, and the location at the theater entrance that had remained open for a century or two, makes these two samples (RTD 9220, 9219, S2 Fig A,B) less secure anchors for the chronological model, and therefore were removed from the model.

Sample RTD 9332 (S2 Fig C) was taken from the “Yellow mortar” underneath the pier between rooms 4403 and 4402. RTD 9347 (S2 Fig D) came from the “Yellow mortar” inside the theater orchestra, after careful cleaning and excavating a hole, 10-20 cm deep, under the surface of the sediment. In the north-east corner of the excavation area, a deep sounding was excavated where the “Yellow mortar” had been cut in the past for the foundation trench of the Western Wall of the temple mount podium. Behind the surface of the ancient cut, charred remains were collected, and sample RTD 9402 (S2 Fig E, F) was dated. RTD 9301 (S2 Fig G) was sampled inside the northern channel.

The channel had been cut into the “Yellow mortar” and covered with grey plaster. Behind this grey plaster, we reached the yellow substance and sampled it for dating.


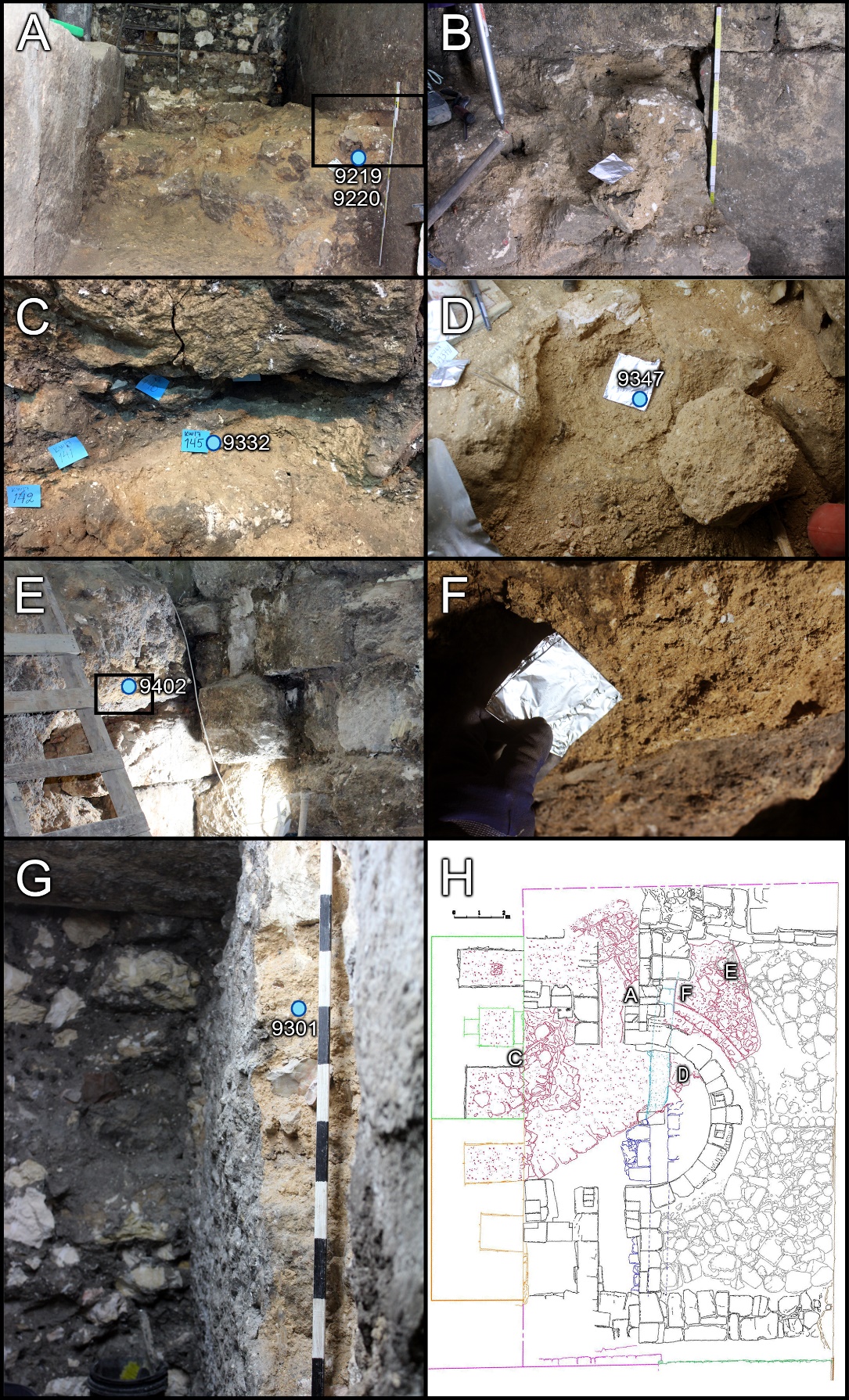


**Fig S2. Radiocarbon sample contexts of Str 8.** “yellow mortar”. ‘B’ and ‘F’ are zoom-in images of the rectangular area marked in ‘A’ and ‘E’, respectively. The red color in H marks wall 4493, e.g. the “yellow mortar”.

**Wilson’s Arch**

The arch consists of two architecturally distinct phases. In both of the constructional phases - the north and the south piers - grey mortar was used between the stones. In room 4402 also white plaster could be identified in several places. The rooms had been filled to about 1.5m height by brown fill material (Fig 2 A, S1 Fig A), having slightly higher clay quantity than calcite. Based on the grinding curves method, the calcite was geogenic, with high crystalline order. The clay component of the sediment is clearly heat un-altered.

*Northern rooms of Wilson’s Arch Str. 7*

The grey mortar between the stones (Fig 2 I, S1 Fig I) has dominant calcite presence, the order of calcite ranging between chalk/ash to plaster. Clay is heat altered with main peak location varying between 1038-1049 cm^-1^. Phosphates presence can be detected in some of the spectra. RTD 9006 came from above the entrance to the room. RTD 9005 came from inside a small niche opposite the entrance to the room (S3 Fig). These parts of the ashlars had not been covered by the Byzantine fill and had stayed clean in an air pocket for the past 1600 years.

Sample RTD 9381 was taken from mortar between the roof and the upper course of stones in the southern wall of room 4401 (S4 Fig).

Samples RTD 9380 and RTD 9379 were taken from grey mortar underneath the pier between rooms (S3 Fig).

*Southern rooms of the Wilson’s Arch Str. 7B*

Two samples were measured from room 4404. RTD 9003 came from the mortar in the northern wall of the room between the top row of stones and the ceiling. RTD 9004 was taken from the mortar between the rooms in the ceiling, close to the eastern wall of the room (S4 Fig). The mortar in these rooms (Fig 2 J, S1 Fig J) is visually like the mortar in the northern rooms. However, the calcite is slightly more ordered (closer to limestone) and the clay main peak is around 1037-1038 cm^-1^, while the peak in the mortar in the north is generally higher.


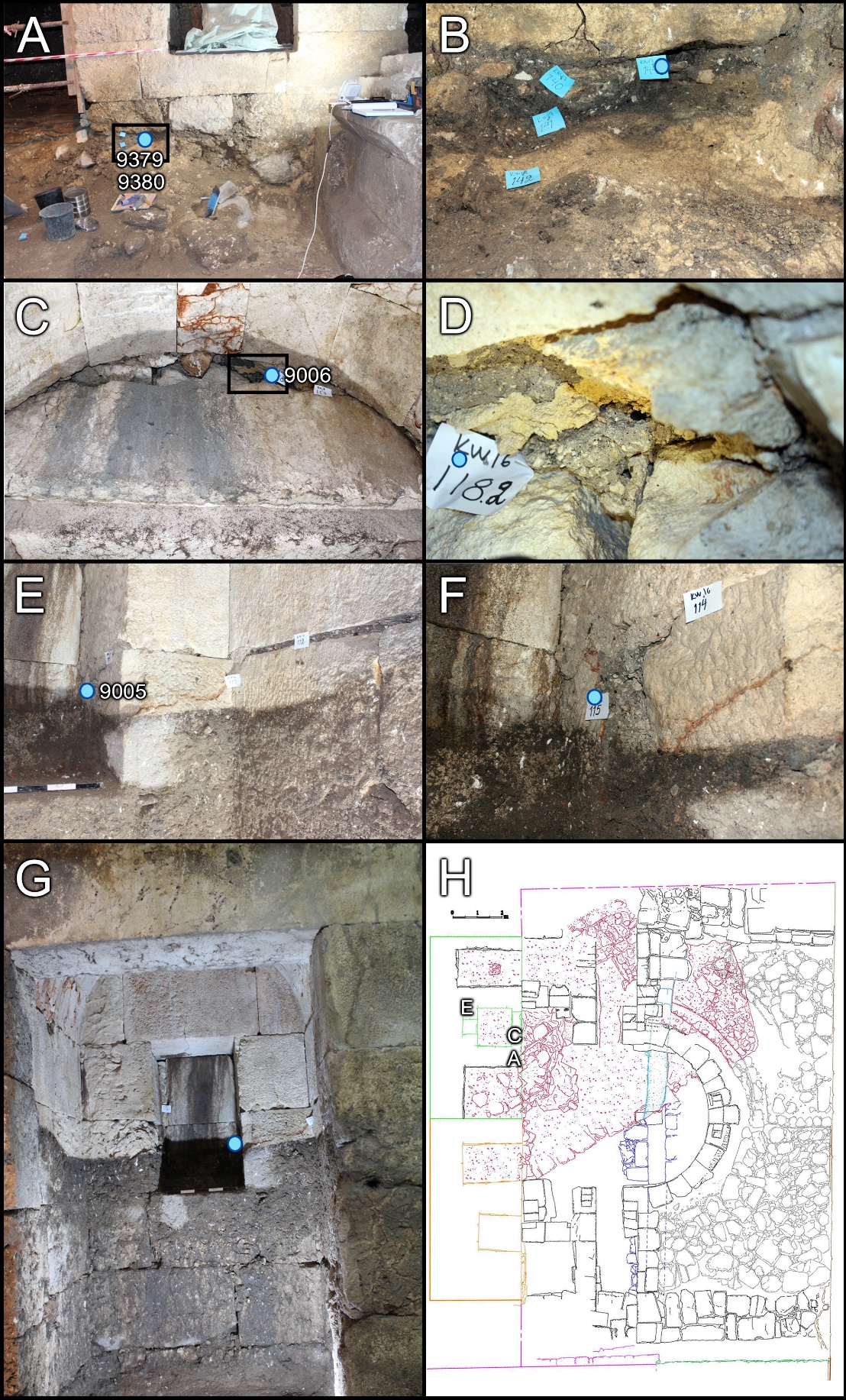


**Fig S3. Radiocarbon sample contexts of Str 7C.** ‘B’ and ‘D’ are zoom-in images of the rectangular in ‘A’ and ‘C’ respectively. ‘E’ and ‘F’ are magnifications of ‘G’.


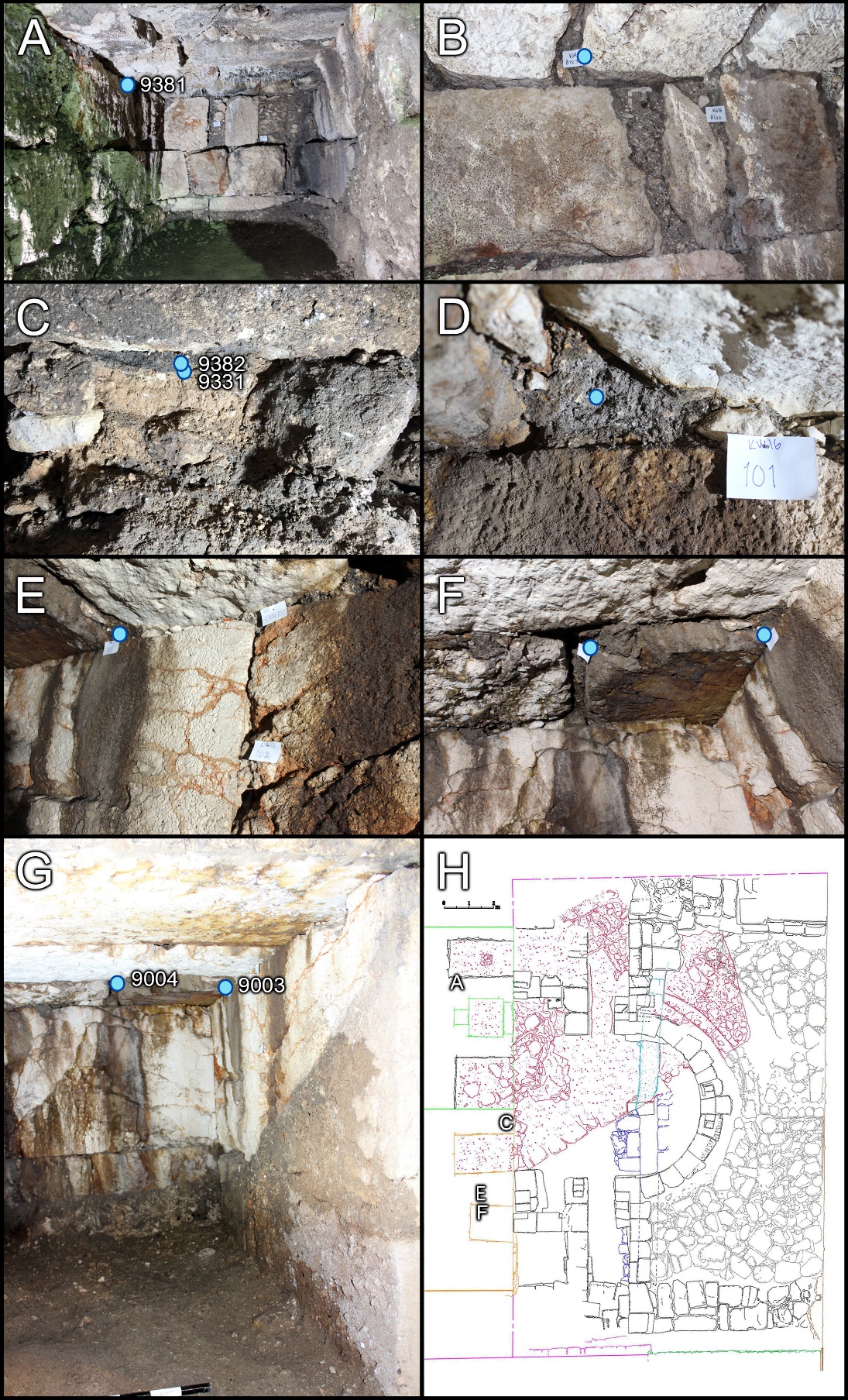


**Fig S4. Radiocarbon sample contexts of Str. 7C and 7B.** ‘B’, ‘D’, and ‘F’ are zoom-in images of ‘A’, ‘E’, and ‘G’, respectively.

**Drainage tunnels underneath the theater**

Two tunnels were built under the theater. The northern channel originally turned east just south of the northern phase of Wilson’s Arch. However, the continuation of the channel was canceled and went out of use, probably at the same time as the northern channel was built. At the connection between the two channels, a flattened stone was found at the level of the expected original flooring of the theater.

*Northern drainage tunnel Str. 7C*

The northern drainage tunnel was partly cut into the “yellow mortar” and covered by c. 2 cm thick grey “plaster”. The major component is pyrogenic calcite (Fig 2 K, S1 Fig K). The clay is altered, peak located at 1040-1045 cm^-1^, and phosphate peaks are present. RTD 9333 and RTD 9334 were taken from this plaster, after the outer surface had been carefully cleaned (S5 Fig).

*Southern drainage tunnel Str. 5C*

The southern channel was built of stones from both sides. However, the plaster did not cover the stones, but was only covering the connections between the stones. The major component of this material is also calcite as lime plaster, based on the grinding curves method. The clay peak is very small, and located lower than in the northern channel, from 1030-1035 cm^-1^. This channel also had an air pocket of c. 50 cm between the top of the channel and the later fill. Top of the fill is grey with medium size stones and lots of charcoal, and the bottom of the fill is brown. The brown fill (Fig 2 C, S1 Fig C) consists of geogenic calcite and non-heat altered clay. RTD 9450 and RTD 9451 were taken from the plaster between the stones, which had been covered by the fill (S5 Fig A, B – before sampling, and D – after sampling).


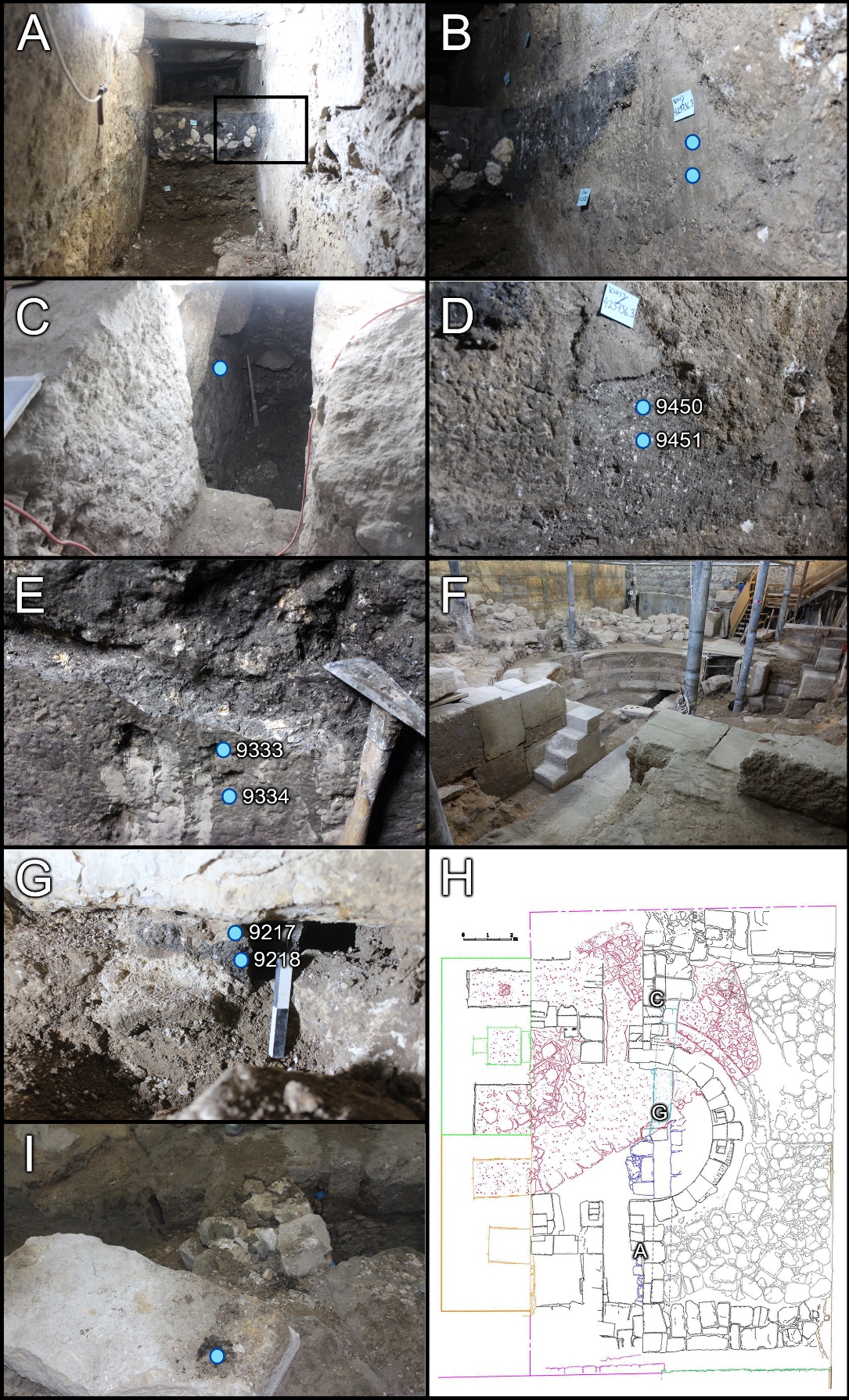


**Fig S5. Radiocarbon sample contexts of Str. 7C and 7B/5C re-plastering.** ‘B’ is the rectangular area in ‘A’. ‘D’, E’, and ‘G’ are zoom-in images of ‘B’, ‘C’, and ‘I’, respectively. ‘F’ is an overview image of the relevant area.

**Theater-like Structure Str. 6**

Locating material for securely dating the theater complex was challenging. No original floors or building materials containing organic material, such as mortars or plasters, could be detected and associated with the monumental architecture. Sediment underlying the theater stones consisted of brown sediment and medium size stones, while only in one place grey sediment rich in charred material was found underlying the stones. As it appears in one location only, it was not regarded as an *in-situ* construction material. However, in order to build the seats, a constructional wall was added to hold in place the fill to elevate the seats. Fortunately, this wall was built using semi-hard grey-brown mortar between the stones. The FTIR spectra of the mortar (Fig 2 H, S1 Fig H) show presence of phosphates, and heated clay. The calcite component of the mortar varies from geogenic to plaster. Soft white lime lumps consisting of plaster were present and could be isolated from the mortar. The FTIR spectra of the brown fill sediment (Fig 2 B, S1 Fig B), covering the wall and the mortar, consisted of relatively higher clay component than in the mortar, the clay being non-heat altered. The calcite in the fill is geogenic. No phosphate presence was detected.

Sample RTD 9373 (B424395.2a) was taken from the uppermost course between two side by side standing large stones. RTD 9389 comes from the western side of the same stone. RTD 9374 (B424397a) comes from the same row, also between two stones standing side by side, but further west from the Western Wall. RTD 9388 comes between two stones one above the other, adjacent to the Western Wall. See S6 Fig for their exact locations.

Inside the fill was built a small wall, most likely functioning to strengthen the structure holding the seats. No mortar was found between these stones, but a brown fill. Two samples were dated from this wall. RTD 9383 came from close to the wall of the theater, while RTD 9378 was sampled closer to the Western Wall (S6 Fig).


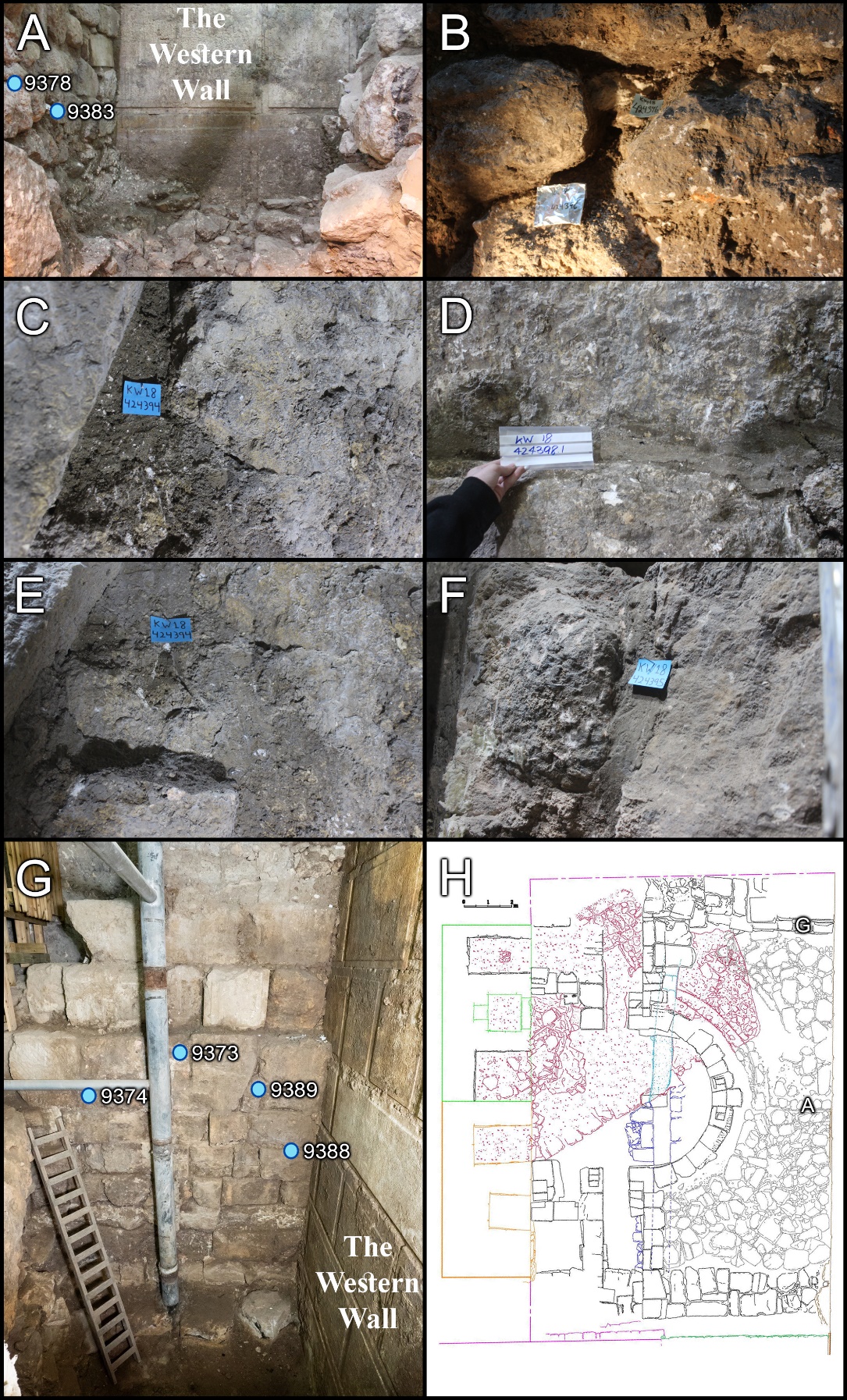


**Fig S6. Radiocarbon sample contexts of Str. 6 (the theater).** ‘B’ is a zoom-in image of ‘A’. ‘E’ and ‘C’ are before and after cleaning the context of RTD 9389, respectively. ‘D’ and ‘F’ are zoom-in images of RTD 9388 and RTD 9373, respectively.

**Late Roman fills above the theater Str. 5B, 4**

The floor of the theater was not found. At the expected floor level and slightly below was found the leveled “Yellow” material and a stone pavement covered with a fill. The theater orchestra was covered by striations of refuse material. The lowest (and earliest) samples measured from material covering the theater were taken from slightly above the lowest course of the theater stones, from a distinct 10 cm thick grey lens/layer, with lots of olive pits. RTD 9099 and RTD 9100 were measured from these olive pits (S7 Fig).

At a higher elevation, the striations continued leaning against the western wall, sloping towards the rooms and the pier of the arch, entering into the rooms and partly filling them. Some of these layers consisted almost purely of olive pits and ash, containing thousands of olive pits. From these olive pit rich layers three directly superimposed samples were dated (S7 Fig). RTD 8815 (B. KW-52) was the lowest sample, and it was taken from a pale grey layer with lots of olive pits. This layer was covered by layer of stones. RTD 8814 (B. KW-48) was taken 60 cm above it from a grey powdery sediment consisting mainly of olive pits, crushed olive pits and ash. This layer was covered by 10 cm of almost white sediment, also full of olives. Above it was another powdery grey layer (Fig 2 D, S1 Fig D) filled with olive pits from which RTD 8813 (B. KW-46) was measured. The main component in the sediment was calcite as ash. Phosphates were present and the clay was heat altered to c. 500-600°C (main peak located at 1038 cm^-1^). The broad peak at 1620 cm^-1^ represents the charcoal presence. The temperature, heating duration and probably reduced oxygen levels seem to have been optimal to produce the excellent preservation of the charred pits.

**North pool Str. 3**

A large cistern built into the byzantine refuse material of str. 5B and 4, was sampled at the north part of the excavation area. Olive pits samples RTD 9129 and RTD 9130 (B20222) were isolated from the white plaster. The main component was calcite (plaster), the small heat altered clay peak was located at 1055 cm^-1^. Small quantity of phosphate was present.


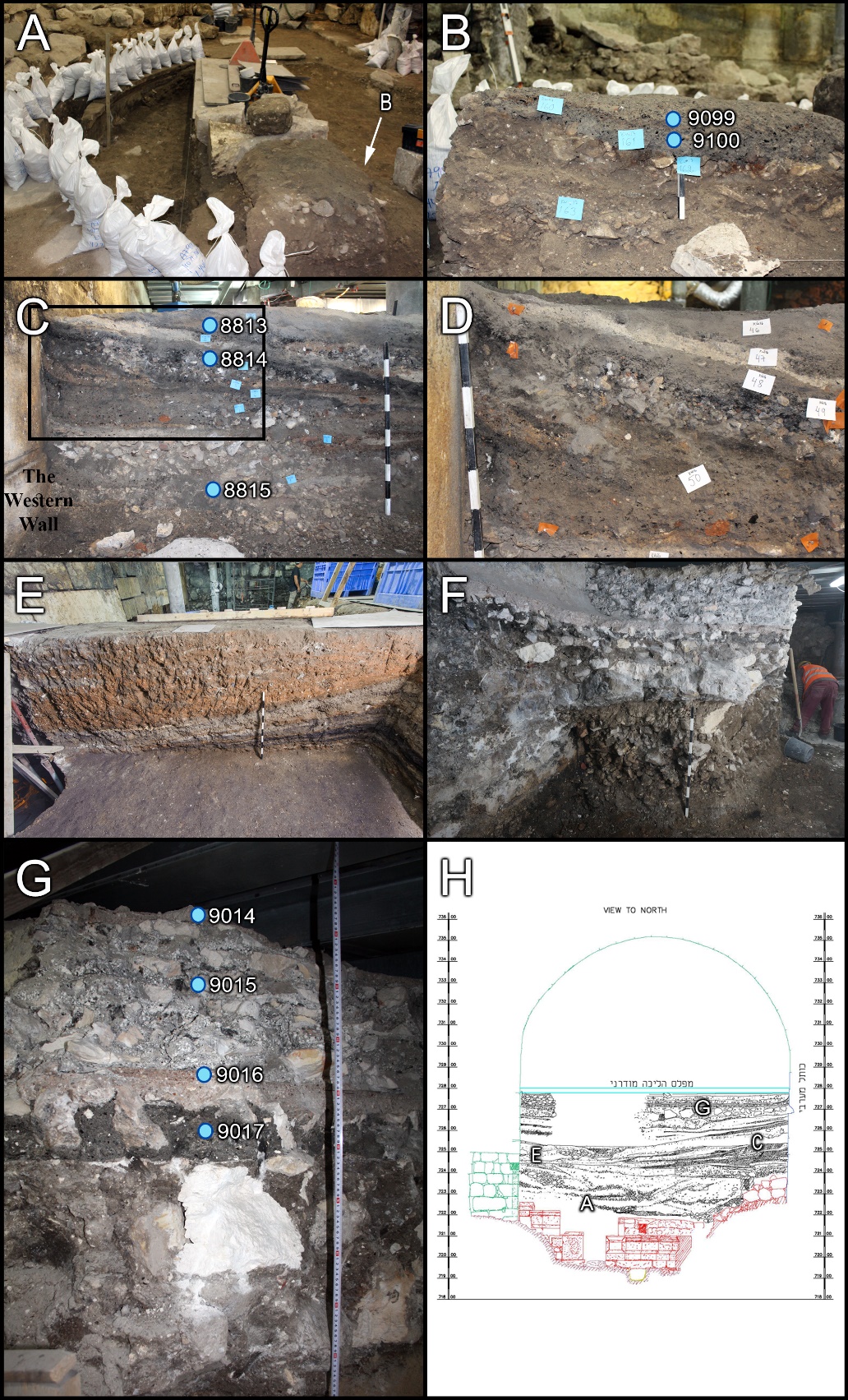


**Fig S7. Radiocarbon sample contexts of the stratified layers of Str. 1,4 and 5B.** ‘A’ is an overview image, showing the location of the section sampled in ‘B’. ‘D’ is a zoom-in image of the rectangular area in ‘C’. ‘E’ is a general image, showing some of the stratified layers in the area. No samples were dated from that view. ‘G’ is a closeup image of the upper part of the section shown in ‘F’.


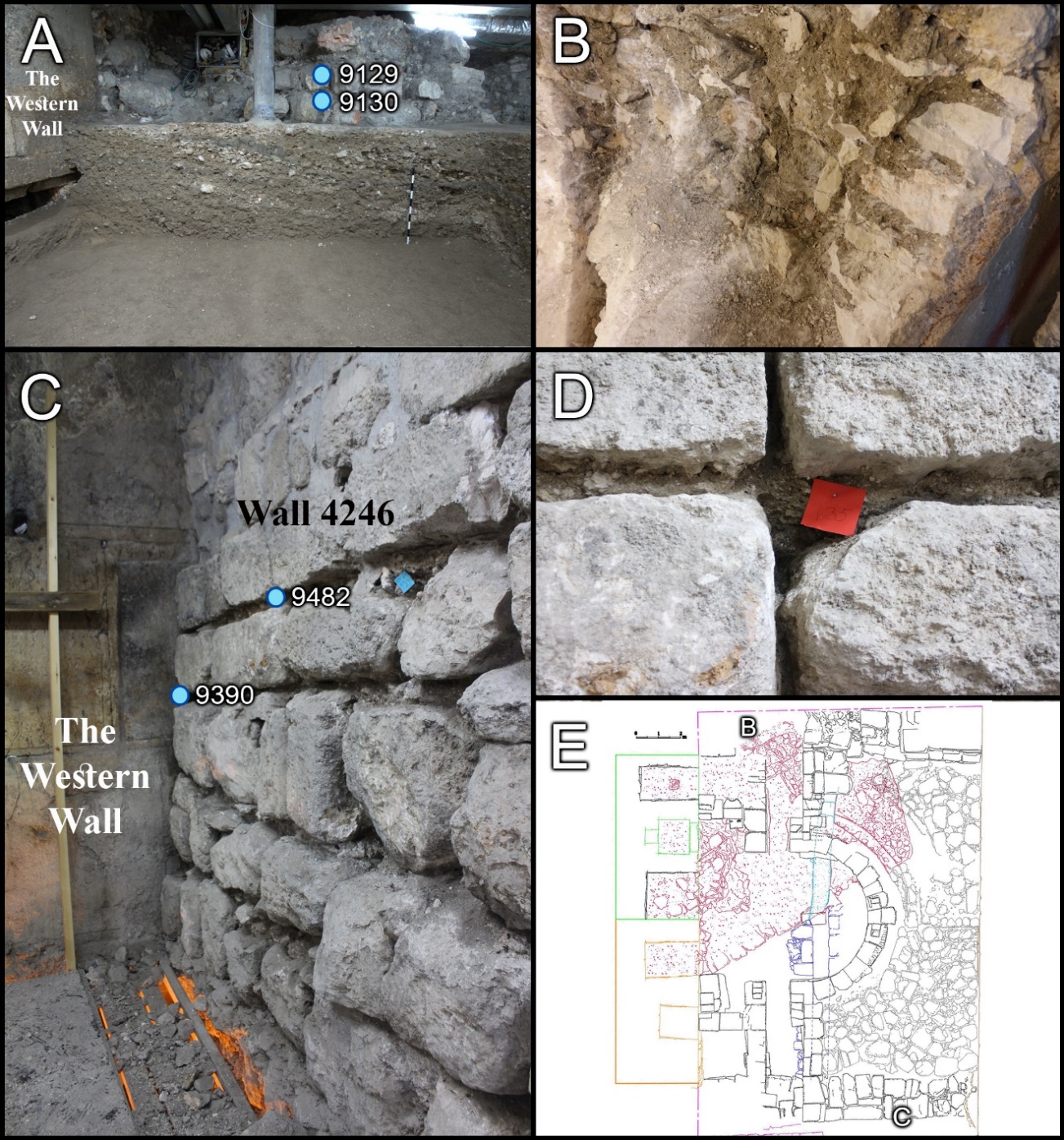


**Fig S8. Radiocarbon sample contexts of Str. 2 wall (A, B) and Str. 3 pool (C, D).** ‘B’ is a zoom-in image of ‘A’. ‘D’ is showing the exact location of RTD 9482.

**Wall built abutting the Western Wall Str. 2**

Wall 4246 abuts the western wall running east to west and also marks the southern border of the excavation area. The wall consists of medium sized roughly hewn stones of various sizes connected with grey mortar. The mortar consists primarily of pyrogenic calcite (ash). The small clay component is heat altered (peak at 1041 cm^-1^) and phosphate presence can be detected. The charred material found inside the mortar consisted primarily of charcoal pieces of *pinus sp*., some pinecone fragments, seeds and cereals. Sample RTD 9390 (B. KW-136) measured from pinecone and sample RTD 9482 (cereal) was taken two meters east from the Western Wall (S8 Fig).

**Sequence of plasters from plaster floors of superimposed pools Str. 1B, 1A**

The lowest layer (L.4217) that was put down probably as a foundation for what will eventually become a series plaster covered pools, consists of roughly 50 cm thick fill. Relatively little charred material was found in this layer, and since there was so little material, we considered it of higher probability that the charred material could be residual, rather than collected and used particularly for the construction purposes. This layer was not dated.

Directly overlying was placed a 10 cm thick dark gray layer (L.4224), cementing together medium sized stones (10 cm), containing large quantities of charred material, and in particular many olive pits. One of the olive pits was dated (RTD 9017, Fig 2 O, S1 Fig O and 7). Above this pool floor a pinkish layer was placed (L.4216) to level and cover the top of the stones, suggesting these layers were constructed at the same time.

The next about 2 cm thick layer (L. 4215) was consisted of a very hard pink plaster. From this plaster, very well preserved stems of grasses were isolated for dating (RTD 9016, Fig 2 O, S1 Fig O and 7). The FTIR showed an additional peak at 3692 cm^-1^, pointing to the presence of brucite Mg(OH)_2_, and the location on the “grinding-curve” plot showed a very well preserved plaster component. Brucite was formed on experiments of heating dolomite stones to form plaster. The presence of the exceptionally well-preserved organic materials is likely to be connected with the unique properties of brucite. In 2006 Hilbertz and Goreau patented the use of brucite as a preservative for wood treatment stating it ”protects the treated wood from degradation by microorganisms, mold, fungi, insects, wood-boring marine organisms, and fire” (16). Our results are in good agreement with this statement as the organic material in our samples survived in excellent state of preservation over 700 years, an exception for un-charred material in this region.

Above this pink plaster layer was placed another thick stone fill of about 40 cm thickness (L.4214), but this time including stones and white plaster with brucite peak, containing large quantity of charred material, from which a seed was dated as sample RTD 9015 (S7 Fig).

The topmost layer (L. 4213) related to the pools is again of pink color with a brucite component. However, this time the organic material used inside the plaster was mainly fibers, most likely flax dated as sample RTD 9014 (Fig 2 N, S1 Fig N and 7).

| **Table S2. Radiocarbon dates, contexts, botanical identification of the dated samples and un-modeled calibrated ranges.** Sample numbers with an asterisk indicate residual samples that were excluded from the model. The δ^13^C is as measured by the AMS system. | | | | | | | | | |
| --- | --- | --- | --- | --- | --- | --- | --- | --- | --- |
| **Sample number** | **Libby age [unCal BP]** | **Stratum** | **Archaeological feature** | **Sample type** | **Basket**  **Locus** | **Elevation [m.a.s.l.]** | **AMS**  **δ^13^C**  **[‰]** | **Cal 1σ** | **Cal 2σ** |
| RTD 9301* | 2999±27 | 8 | Wall 4493 “yellow mortar” behind the mortar of the western wall of northern channel | Seed (cereal) | B.424313.2  L.4547 | 721.55 upper, 720.25 lower | -23.34 | 1280BC (65.1%) 1195BC  1141BC (3.1%) 1134BC | 1375BC (4.5%) 1353BC 1302BC (90.9%) 1127BC |
| RTD 9347 | 2120±26 |  | "Yellow mortar" under theater podium | Seed  (cf. almond) | B.423742.4  L.4525 | 719.24 | -18.97 | 195BC (68.2%) 108BC | 342BC (2.4%) 328BC 205BC (93.0%) 53BC |
| RTD 9402 | 2048±31 |  | "Yellow mortar" next to foundation trench of Western Wall | Charcoal (moraceae) | B.424435.2  L.4429 | 721.51 upper, 721.04 lower | -19.55 | 102BC (68.2%) 1AD | 166BC (95.4%) 22AD |
| RTD 9332 | 2048±27 |  | "Yellow mortar" under pier between rooms 4403 and 4402 | Seed | B.KW145  L.4465 | 721.9 | -24.73 | 96BC (60.0%) 19BC  13BC (8.2%) 1AD | 164BC (10.1%) 128BC 121BC (85.3%) 20AD |
| RTD 9219 | 1994±42 | Unclear | "Yellow mortar" at the northern entrance to the theater | Seed (barley) | B.423581.2  L.4501 | 720.8 upper, 719.7 lower | -25.85 | 41BC (68.2%) 53AD | 107BC (94.3%) 87AD 106AD (1.1%) 120AD |
| RTD 9220 | 1974±24 |  |  | Seed |  | 4501 | -23.13 | 3AD (68.2%) 59AD | 40BC (95.4%) 74AD |
| RTD 9381 | 2019±26 | 7C | Wilson's arch northern pier room 4401; grey mortar between top row of stones and ceiling | Seed  (cf. wheat) | B.KW123.2  L.4401 Room | 724.45 | -22.82 | 46BC (68.2%) 16AD | 92BC (95.4%) 54AD |
| RTD 9005 | 2005±23 |  | Wilson's arch northern pier room 4402; grey mortar between stones above height of the fill covering the room | Seed  (grape pip) | B.KW115  L.4402 Room | 724.6 | -29.25 | 40BC (68.2%) 21AD | 51BC (95.4%) 57AD |
| RTD 9006 | 2000±30 |  | Wilson's arch northern pier room 4402; grey mortar above the entrance to the room | Seed or charcoal | B.KW118  L.4402 Room | 724.5 | -22.95 | 41BC (63.7%) 27AD  42AD (4.5%) 47AD | 88BC (1.4%) 77BC  56BC (94.0%) 72AD |
| RTD 9379 | 1984±25 |  | Wilson's arch northern pier between rooms 4403 and 4402; grey mortar under pier | Seed (olive) | B.KW144A  L.4465 | 721.9 | -19.48 | 21BC (7.9%) 11BC  2BC (60.3%) 55AD | 42BC (95.4%) 66AD |
| RTD 9380 | 1981±26 |  | Wilson's arch northern pier between rooms 4403 and 4402; grey mortar under pier | Seed (olive) | B.KW144B  L.4465 | 721.9 | -21.57 | 20BC (6.5%) 12BC  1BC (61.7%) 58AD | 42BC (95.4%) 70AD |
| RTD 9333 | 2007±26 |  | Channel northern drainage grey plaster covering the channel wall | Seed  (grape pip) | B.423737  L.4504 | 719.0 | -25.71 | 41BC (68.2%) 21AD | 85BC (0.9%) 79BC  55BC (94.5%) 62AD |
| RTD 9334 | 1977±27 |  | Channel northern drainage grey plaster covering the channel wall | Seed | B.423737.4  L.4504 | 719.0 | -23.60 | 18BC (2.9%) 14BC  1AD (65.3%) 62AD | 42BC (95.4%) 75AD |
| RTD 9217 | 1981±23 | Unclear | Under Theater/north channel; grey mortar under stone | Seed  (grape pip) | B.4023619.3  L.4506 | 719.4 | -24.59 | 19BC (4.3%) 13BC  1AD (63.9%) 56AD | 41BC (95.4%) 68AD |
| RTD 9218 | 1959±24 |  |  | Seed (cereal) | B.423619.3  L.4506 | 719.4 | -28.16 | 20AD (68.2%) 71AD | 38BC (6.2%) 9BC  4BC (88.4%) 85AD  110AD (0.8%) 115AD |
| RTD 9331* | 2052±25 | 7B | Wilson's arch southern pier between rooms 4404 and 4403; grey mortar under pier | Seed (cereal) | B.KW154  L.4525 | 721.0 | -24.32 | 109BC (57.9%) 36BC  31BC (5.0%) 21BC  11BC (5.4%) 2BC | 166BC (95.4%) 5AD |
| RTD 9382 | 1992±25 |  | Wilson's arch southern pier between rooms 4404 and 4403; grey mortar under pier | Seed (olive) |  |  | -25.67 | 37BC (8.8%) 28BC  23BC (13.8%) 10BC  3BC (35.2%) 28AD  39AD (10.3%) 49AD | 46BC (95.4%) 65AD |
| RTD 9004 | 1959±46 |  | Wilson's arch southern pier room 4404; grey mortar between the stones in the ceiling | Seed (cereal) | B.KW103.2  L.4404 Room | 724.4 | -22.74 | 36BC (2.1%) 31BC  21BC (4.7%) 11BC  2BC (61.5%) 83AD | 90BC (0.9%) 74BC  58BC (94.5%) 135AD |
| RTD 9003 | 1947±22 |  | Wilson's arch southern pier; room 4404; grey mortar between the upper row of stones and ceiling | Seed  (grape pip) | B.KW101.2  L.4404 | 724.4 | -23.38 | 25AD (68.2%) 78AD | 2AD (88.4%) 90AD  99AD (7.0%) 124AD |
| RTD 9389 | 1915±25 | 6 | Theater north wall; grey-brown mortar between stones of the wall | Seed | B.424394.1  L.W4451 | 721.8 | -23.97 | 65AD (36.2%) 92AD  98AD (32.0%) 124AD | 24AD (95.4%) 134AD |
| RTD 9373 | 1908±23 |  |  | Seed (olive) | B.424395.2a  L.W4451 | 722.3 | -23.28 | 72AD (68.2%) 125AD | 27AD (2.1%) 41AD  48AD (93.3%) 135AD |
| RTD 9374 | 1876±23 |  |  | Twig  (cf. juniper) | B.424397a  L.W4451 | 721.9 | -23.47 | 78AD (60.4%) 140AD 160AD (2.3%) 165AD 197AD (5.5%) 208AD | 74AD (95.4%) 215AD |
| RTD 9388 | 1874±25 |  |  | Charcoal  (cf reed) | B.424398.1a  L.W4451 | 721.0 | -22.35 | 79AD (57.0%) 141AD 158AD (4.2%) 166AD 196AD (7.1%) 208AD | 74AD (95.4%) 219AD |
| RTD 9378* | 2399±27 |  | Theater under seats; brown fill under theater seats | Seed (pulse) | B.424346  L.4409 | 719.6 | -24.91 | 507BC (2.6%) 503BC 490BC (65.6%) 406BC | 728BC (1.8%) 716BC 707BC (2.3%) 694BC 542BC (91.3%) 400BC |
| RTD 9383 | 1828±25 |  | Str. 6 Theater under seats; brown fill under theater seats | Seed (olive) | B.424347  L.4409 | 719.6 | -20.92 | 139AD (53.0%) 198AD 204AD (15.2%) 221AD | 90AD (1.1%) 100AD  124AD (94.3%) 250AD |
| RTD 9450 | 1853±28 | 5C | Channel southern re-plastered, grey plaster covering cracks between the stones | Seed | B.423736.3  L.4513 | 718.7 | -22.17 | 127AD (68.2%) 215AD | 85AD (95.4%) 233AD |
| RTD 9451 | 1751±31 |  |  | Twig |  |  | -15.49 | 244AD (17.1%) 265AD 272AD (51.1%) 332AD | 216AD (95.4%) 387AD |
| RTD 9099 | 1781±26 | 5B | Fill above theater | Seed (olive) | B.KW161  L.4448 | 721.6 | -20.74 | 218AD (35.2%) 260AD 280AD (33.0%) 325AD | 138AD (16.3%) 200AD 206AD (79.1%) 333AD |
| RTD 9100 | 1770±26 |  |  | Seed (olive) | B.KW161a  L.4448 | 721.4 | -21.89 | 235AD (25.3%) 260AD 279AD (42.9%) 326AD | 142AD (7.4%) 196AD 209AD (88.0%) 342AD |
| RTD 8814 | 1797±21 | 4 | Fill middle grey with ash full of olive pits | Seed (olive) | B.KW48  L.4293 | 725.0 | -19.84 | 144AD (4.2%) 152AD 169AD (15.4%) 194AD 210AD (43.8%) 253AD 304AD (4.8%) 313AD | 134AD (83.9%) 258AD 285AD (0.5%) 287AD  296AD (10.9%) 322AD |
| RTD 8815 | 1759±22 |  | Fill lowest grey with ash many olive pits | Seed (olive) | B.KW52  L.4295 | 724.9 | -20.04 | 244AD (18.1%) 260AD 280AD (50.1%) 325AD | 221AD (95.4%) 347AD |
| RTD 8813 | 1750±21 |  | Fill highest grey with ash full of olive pits | Seed (olive) | B.KW46  L.4291 | 725.3 | -20.45 | 249AD (13.4%) 263AD 276AD (54.8%) 329AD | 235AD (94.6%) 348AD 371AD (0.8%) 377AD |
| RTD 9130 | 1574±17 | 3 | North pool; from white plaster | Seed (barley) | B.420221  L.4230 | 726.0 | -22.33 | 429AD (7.0%) 437AD 445AD (24.9%) 473AD 486AD (9.7%) 497AD 505AD (26.7%) 535AD | 425AD (95.4%) 539AD |
| RTD 9129 | 1529±17 |  | North pool; from white plaster | Seed (olive) | B.420222  L.4226 | 726.0 | -23.20 | 437AD (5.8%) 445AD 473AD (10.9%) 486AD 535AD (51.4%) 570AD | 430AD (34.7%) 493AD 530AD (60.7%) 593AD |
| RTD 9390 | 1436±24 | 2 | South wall; soft grey mortar full of ash between the Western Wall and the south wall | Pinecone | B.KW136a1  L.4246 | 727.0 | -24.50 | 605AD (68.2%) 645AD | 578AD (95.4%) 653AD |
| RTD 9482 | 1384±23 |  | South wall; soft grey mortar full of ash between the stones | Seed (cereal) | B.KW135  L.4246 | 727.0 | -22.41 | 641AD (68.2%) 663AD | 613AD (95.4%) 670AD |
| RTD 9017 | 702±26 | 1B | Pool 50cm thick mortar with stones and charred material | Seed (olive) | B.KW200  L.4224 | 727.2 | -24.30 | 1271AD (68.2%) 1296AD | 1262AD (83.3%) 1305AD  1365AD (12.1%) 1385AD |
| RTD 9016 | 558±33 |  | Pool 4cm thick pink plaster with grasses | Cellulose (grasses) | B.420138  L.4215 | 727.2 | -31.24 | 1320AD (33.4%) 1350AD  1391AD (34.8%) 1419AD | 1306AD (49.3%) 1364AD 1385AD (46.1%) 1432AD |
| RTD 9015 | 618±35 | 1A | Pool 30 cm thick grey mortar with stones with charred material | Seed | B.420137  L.4214 | 727.6 upper, 727.3  lower | -28.04 | 1299AD (26.7%) 1325AD 1345AD (26.8%) 1371AD 1379AD (14.8%) 1394AD | 1290AD (95.4%) 1404AD |
| RTD 9014 | 577±35 |  | Pool 2cm thick pink plaster with flax fibers | Cellulose (flax Fibers) | B.420136  L.4213 | 727.6 | -27.84 | 1315AD (44.8%) 1356AD 1389AD (23.4%) 1410AD | 1299AD (61.8%) 1370AD 1380AD (33.6%) 1422AD |

**Table S3. Modeled (posterior) dates comparison between the ‘Standard Model’ (in white) after omitting the three outliers, and the ‘Outlier Model’ which includes them (in green).** Of the latter only one example is shown of the several slightly different obtained. See below for the models code. The outliers are marked in yellow. The dates with the ~10 years shift are in bold.

| **Model Stratum** | **Sample Lab Code** | **Standard, Model**  **Modelled ranges (BC/AD)**  **68.2%** | **Agreement of Sample**  (**Amodel = 90.0**) | **Outlier Model Modelled ranges (BC/AD)**  **68.2%** | **Agreement of Sample** (**Amodel = 32.2**) | Posterior |
| --- | --- | --- | --- | --- | --- | --- |
| Str 8: Wall with “yellow mortar“ | RTD-9301 |  |  | 119-47 | 8.3 | 97 |
|  | RTD-9347 | 114-53 BC | 71.2 | 121-54 BC | 78.4 | 1 |
|  | RTD-9332 | 101-46 BC | 105.4 | 105-47 BC | 106.3 | 1 |
|  | RTD-9402 | 102-46 BC | 108.8 | 107-48 BC | 109.9 | 1 |
| Str 7C: Northern pier of arch | RTD-9381 | **22BC-20AD** | 112.4 | **36BC-10 AD** | 127 | 0 |
|  | RTD-9005 | **22BC-20AD** | 116.3 | **36BC-10 AD** | 124.1 | 0 |
|  | RTD-9006 | **22BC-20AD** | 121.5 | **36BC-10 AD** | 126.5 | 0 |
|  | RTD-9379 | **22BC-20AD** | 102.2 | **37BC-11 AD** | 96.8 | 0 |
|  | RTD-9380 | **22BC-20AD** | 97.6 | **37BC-11 AD** | 91.5 | 0 |
| Str 7C: Northern drainage channel | RTD-9333 | **23 BC-20 AD** | 117.5 | **36BC-10 AD** | 126.6 | 0 |
|  | RTD-9334 | **22BC-20AD** | 90.7 | **37BC-10 AD** | 83.4 | 0 |
| Str 7B: Southern pier of arch | RTD-9331 |  |  | 4BC-50 AD | 20.9 | 8 |
|  | RTD-9382 | **27-59AD** | 88.5 | **12-51 AD** | 110.2 | 0 |
|  | RTD-9004 | **29-61 AD** | 136.7 | **14-52 AD** | 136 | 0 |
|  | RTD-9003 | **29-61 AD** | 112.5 | **18-55 AD** | 96 | 0 |
| Str 6: Theater north wall | RTD-9389 | 96-129 AD | 105.9 | 95-128 AD | 112.1 | 0 |
|  | RTD-9373 | 96-129 AD | 108.7 | 96-128 AD | 114.8 | 0 |
|  | RTD-9374 | 95-133 AD | 128.5 | 95-131 AD | 135.6 | 0 |
|  | RTD-9388 | 95-133 AD | 128.1 | 95-131 AD | 134.7 | 0 |
| Str 6: Theater seats | RTD-9378 |  |  | 118-155 AD | 5.6 | 100 |
|  | RTD-9383 | 124-155 AD | 80.6 | 125-159 AD | 87.5 | 1 |
| Str 5C: South channel replaster | RTD-9450 | 195-238 AD | 88.9 | 196-238 AD | 91.2 | 1 |
|  | RTD-9451 | 217-266 AD | 62.3 | 217-267 AD | 64.6 | 2 |
| Str 5B: Fill sealing theater | RTD-9099,9100 R_Combine | 239-306 AD | 101.1 | 239-306 AD | 106.1 | 0 |
| Str 4: Fill Late Roman | RTD-8815 | 291-326 AD | 114.4 | 291-326 AD | 119.7 | 0 |
|  | RTD-8814 | 294-324 AD | 60.4 | 294-324 AD | 62.8 | 1 |
|  | RTD-8813 | 289-328 AD | 110.5 | 289-328 AD | 115.7 | 0 |
| Str 3: North pool | RTD-9130 | 435-537 AD | 99.4 | 437-537 AD | 103.7 | 0 |
|  | RTD-9129 | 435-555 AD | 79 | 435-555 AD | 83.4 | 1 |
| Str 2: South wall | RTD-9390 | 617-649 AD | 104.9 | 617-649 AD | 109.5 | 0 |
|  | RTD-9482 | 642-663 AD | 93.7 | 639-662 AD | 97.8 | 0 |
| Str 1B: Lower pool | RTD-9017 | 1275-1295 AD | 102.7 | 1275-1295 AD | 107.5 | 0 |
|  | RTD-9016 | 1307-1340 AD | 78 | 1308-1340 AD | 81.7 | 2 |
| Str 1a: Upper pool | RTD-9015 | 1330-1397 AD | 99.4 | 1335-1398 AD | 103.8 | 0 |
|  | RTD-9014 | 1327-1402 AD | 101.8 | 1328-1404 AD | 106 | 0 |

**S6 The OxCal model code (the ‘Standard Model’)**

Plot()

{

Sequence("Wilson's Arch")

{

Boundary("Start Str 8");

Phase("Str 8: Wall with 'yellow mortar'")

{

R_Date("RTD-9347", 2120, 26);

R_Date("RTD-9332", 2048, 27);

R_Date("RTD-9402", 2048, 31);

};

Boundary("End Str 8");

Boundary("Start Str 7");

Phase()

{

Sequence()

{

Boundary("Start 1");

Phase("Str 7C: Northern pier of arch")

{

R_Date("RTD-9381", 2019, 26);

R_Date("RTD-9005", 2005, 23);

R_Date("RTD-9006", 2000, 30);

R_Date("RTD-9379", 1984, 25);

R_Date("RTD-9380", 1981, 26);

};

Boundary("End 1");

};

Sequence()

{

Boundary("Start 2");

Phase("Str 7C: Northern drainage channel")

{

R_Date("RTD-9333", 2007, 26);

R_Date("RTD-9334", 1977, 27);

};

Boundary("End 2");

};

};

Boundary("End Str 7C");

Boundary("Start Str 7B");

Phase("Str 7B: Southern pier of arch")

{

R_Date("RTD-9382", 1992, 25);

R_Date("RTD-9004", 1959, 46);

R_Date("RTD-9003", 1947, 22);

};

Boundary("End Str 7B");

Boundary("Start Str 6");

Sequence("Str 6: Theater")

{

Phase("Str 6: Theater north wall")

{

R_Date("RTD-9389", 1915, 25);

R_Date("RTD-9373", 1908, 23);

R_Date("RTD-9374", 1876, 23);

R_Date("RTD-9388", 1874, 25);

};

Phase("Str 6: Theater seats")

{

R_Date("RTD-9383", 1828, 25);

};

};

Boundary("End Str 6");

Boundary("Start Str 5");

Phase("Str 5C: South channel replaster")

{

R_Date("RTD-9450", 1853, 28);

R_Date("RTD-9451", 1751, 25);

};

Boundary("Transition Str 5 early/late");

Phase("Str 5B: Fill sealing theater")

{

R_Combine("RTD-9099,9100")

{

R_Date("RTD-9099", 1781, 26);

R_Date("RTD-9100", 1770, 26);

};

};

Boundary("Transition Str 5A/4");

Phase("Str 4: Fill late roman")

{

R_Date("RTD-8815", 1759, 22);

R_Date("RTD-8814", 1797, 21);

R_Date("RTD-8813", 1750, 21);

};

Boundary("End Str 4");

Boundary("Start Str 3");

Phase("Str 3: North pool")

{

R_Date("RTD-9130", 1574, 17);

R_Date("RTD-9129", 1529, 17);

};

Boundary("End Str 3");

Boundary("Start Str 2");

Phase("Str 2: South wall")

{

R_Date("RTD-9390", 1436, 24);

R_Date("RTD-9482", 1384, 23);

};

Boundary("End Str 2");

Boundary("Start Str 1B");

Phase("Str 1B: Lower pool")

{

R_Date("RTD-9017", 702, 26);

R_Date("RTD-9016", 558, 33);

};

Boundary("Transition Str 1b/1a");

Phase("Str 1a: Upper pool")

{

R_Date("RTD-9015", 618, 35);

R_Date("RTD-9014", 577, 35);

};

Boundary("End Str 1");

};

};

**S7 The OxCal outlier model code (the ‘Outlier Model’)**

Plot()

{

Outlier_Model("General",T(5),U(0,4),"t");

Sequence("Wilson's Arch")

{

Boundary("Start Str 8");

Phase("Str 8: Wall with 'yellow mortar'")

{

R_Date("9301", 2999, 27)

{

Outlier(.05);

};

R_Date("RTD-9347", 2120, 26)

{

Outlier(.05);

};

R_Date("RTD-9332", 2048, 27)

{

Outlier(.05);

};

R_Date("RTD-9402", 2048, 31)

{

Outlier(.05);

};

};

Boundary("End Str 8");

Boundary("Start Str 7");

Phase()

{

Sequence()

{

Boundary("Start 1");

Phase("Str 7C: Northern pier of arch")

{

R_Date("RTD-9381", 2019, 26)

{

Outlier(.05);

};

R_Date("RTD-9005", 2005, 23)

{

Outlier(.05);

};

R_Date("RTD-9006", 2000, 30)

{

Outlier(.05);

};

R_Date("RTD-9379", 1984, 25)

{

Outlier(.05);

};

R_Date("RTD-9380", 1981, 26)

{

Outlier(.05);

};

};

Boundary("End 1");

};

Sequence()

{

Boundary("Start 2");

Phase("Str 7C: Northern drainage channel")

{

R_Date("RTD-9333", 2007, 26)

{

Outlier(.05);

};

R_Date("RTD-9334", 1977, 27)

{

Outlier(.05);

};

};

Boundary("End 2");

};

};

Boundary("End Str 7C");

Boundary("Start Str 7B");

Phase("Str 7B: Southern pier of arch")

{

R_Date("9331", 2052, 25)

{

Outlier(.05);

};

R_Date("RTD-9382", 1992, 25)

{

Outlier(.05);

};

R_Date("RTD-9004", 1959, 46)

{

Outlier(.05);

};

R_Date("RTD-9003", 1947, 22)

{

Outlier(.05);

};

};

Boundary("End Str 7B");

Boundary("Start Str 6");

Sequence("Str 6: Theater")

{

Phase("Str 6: Theater north wall")

{

R_Date("RTD-9389", 1915, 25)

{

Outlier(.05);

};

R_Date("RTD-9373", 1908, 23)

{

Outlier(.05);

};

R_Date("RTD-9374", 1876, 23)

{

Outlier(.05);

};

R_Date("RTD-9388", 1874, 25)

{

Outlier(.05);

};

};

Phase("Str 6: Theater seats")

{

R_Date("9378", 2399, 27)

{

Outlier(.05);

};

R_Date("RTD-9383", 1828, 25)

{

Outlier(.05);

};

};

};

Boundary("End Str 6");

Boundary("Start Str 5");

Phase("Str 5C: South channel replaster")

{

R_Date("RTD-9450", 1853, 28)

{

Outlier(.05);

};

R_Date("RTD-9451", 1751, 25)

{

Outlier(.05);

};

};

Boundary("Transition Str 5 early/late");

Phase("Str 5B: Fill sealing theater")

{

R_Combine("RTD-9099,9100")

{

Outlier(.05);

R_Date("RTD-9099", 1781, 26);

R_Date("RTD-9100", 1770, 26);

};

};

Boundary("Transition Str 5A/4");

Phase("Str 4: Fill late roman")

{

R_Date("RTD-8815", 1759, 22)

{

Outlier(.05);

};

R_Date("RTD-8814", 1797, 21)

{

Outlier(.05);

};

R_Date("RTD-8813", 1750, 21)

{

Outlier(.05);

};

};

Boundary("End Str 4");

Boundary("Start Str 3");

Phase("Str 3: North pool")

{

R_Date("RTD-9130", 1574, 17)

{

Outlier(.05);

};

R_Date("RTD-9129", 1529, 17)

{

Outlier(.05);

};

};

Boundary("End Str 3");

Boundary("Start Str 2");

Phase("Str 2: South wall")

{

R_Date("RTD-9390", 1436, 24)

{

Outlier(.05);

};

R_Date("RTD-9482", 1384, 23)

{

Outlier(.05);

};

};

Boundary("End Str 2");

Boundary("Start Str 1B");

Phase("Str 1B: Lower pool")

{

R_Date("RTD-9017", 702, 26)

{

Outlier(.05);

};

R_Date("RTD-9016", 558, 33)

{

Outlier(.05);

};

};

Boundary("Transition Str 1b/1a");

Phase("Str 1a: Upper pool")

{

R_Date("RTD-9015", 618, 35)

{

Outlier(.05);

};

R_Date("RTD-9014", 577, 35)

{

Outlier(.05);

};

};

Boundary("End Str 1");

};

};
